# Cellular stress induces erythrocyte assembly on intravascular von Willebrand factor strings and promotes microangiopathy

**DOI:** 10.1038/s41598-018-28961-2

**Published:** 2018-07-19

**Authors:** Jan P. Nicolay, Verena Thorn, Christoph Daniel, Kerstin Amann, Balasaheb Siraskar, Florian Lang, Carina Hillgruber, Tobias Goerge, Stefan Hoffmann, Christian Gorzelanny, Volker Huck, Christian Mess, Tobias Obser, Reinhard Schneppenheim, Ingrid Fleming, Matthias F. Schneider, Stefan W. Schneider

**Affiliations:** 1 Department of Dermatology, Venereology and Allergy, University Medical Center Mannheim, Ruprecht-Karls-University of Heidelberg, Mannheim, Germany; 2 Division of Immunogenetics, German Cancer Research Center (DKFZ), Heidelberg, Germany; 3 Department of Nephropathology, Friedrich-Alexander-University (FAU) Erlangen-Nürnberg, Erlangen, Germany; 4 Department of Physiology, University of Tübingen, Tübingen, Germany; 5 Department of Dermatology, University Hospital Münster, Münster, Germany; 6 Institute of Plant Biology and Biotechnology (IBBP), Westfälische Wilhelms-Universität Münster, Münster, Germany; 7 Department of Pediatric Hematology and Oncology, University Medical Center Hamburg-Eppendorf, Hamburg, Germany; 8 Institute for Vascular Signalling, Centre for Molecular Medicine, Goethe University, Frankfurt am Main, Germany; 9 Department of Physics, Technical University of Dortmund, Dortmund, Germany; 10 Department of Dermatology and Venerology, University Medical Center Hamburg-Eppendorf, Hamburg, Germany

## Abstract

Microangiopathy with subsequent organ damage represents a major complication in several diseases. The mechanisms leading to microvascular occlusion include von Willebrand factor (VWF), notably the formation of ultra-large von Willebrand factor fibers (ULVWFs) and platelet aggregation. To date, the contribution of erythrocytes to vascular occlusion is incompletely clarified. We investigated the platelet-independent interaction between stressed erythrocytes and ULVWFs and its consequences for microcirculation and organ function under dynamic conditions. In response to shear stress, erythrocytes interacted strongly with VWF to initiate the formation of ULVWF/erythrocyte aggregates via the binding of Annexin V to the VWF A1 domain. VWF-erythrocyte adhesion was attenuated by heparin and the VWF-specific protease ADAMTS13. In an *in vivo* model of renal ischemia/reperfusion injury, erythrocytes adhered to capillaries of wild-type but not VWF-deficient mice and later resulted in less renal damage. *In vivo* imaging in mice confirmed the adhesion of stressed erythrocytes to the vessel wall. Moreover, enhanced eryptosis rates and increased VWF binding were detected in blood samples from patients with chronic renal failure. Our study demonstrates that stressed erythrocytes have a pronounced binding affinity to ULVWFs. The discovered mechanisms suggest that erythrocytes are essential for the pathogenesis of microangiopathies and renal damage by actively binding to ULVWFs.

## Introduction

Tissue dysfunction and organ damage caused by microangiopathies are major factors in the morbidity and mortality of patients with a variety of diseases, including thrombotic thrombocytopenic purpura (TTP), hemolytic uremic syndrome (HUS), connective tissue disease, sepsis and diabetes^1,2^. Renal damage and subsequent kidney failure is a typical and severe complication of microangiopathy. In addition to inflammatory or immune complex-mediated mechanisms of vascular damage, microangiopathic damage can also result from the adhesion of corpuscular components to the endothelium, leading to vascular obstruction. In TTP patients, this effect is supposed to be mainly mediated by the formation of endothelial-derived ultra-large von Willebrand factor fibers (ULVWFs) and platelet aggregation^3,4^. These ULVWFs were recently demonstrated to also exist in tumor microvessels, as shown in mice and human tissues^5,6^. Under different pathological conditions, such as Wilson’s disease^7^, diabetes^8^, Alzheimer’s disease^9^, sickle cell disease^10^ and HUS^11–13^, erythrocytes undergo eryptosis^14^, an apoptosis-like cell death, and adhere to the vascular endothelium. However, the mechanisms involved in erythrocyte-endothelial adhesion are incompletely understood. A variety of molecules have been proposed as possible mediators^15–17^, but they do not completely account for the observed microangiopathic effect. In addition, there are only sparse *in vivo* data supporting these candidates. We propose VWF, a high-molecular-weight glycoprotein, as a new candidate molecule that contributes to erythrocyte endothelial adhesion and thereby promotes microvascular occlusion. VWF is known to form highly adhesive large fibrillar polymers in a highly dynamic process under shear flow conditions^18^. The glycoprotein is stored in Weibel-Palade bodies (WPBs) within endothelial cells (ECs) and is released into the vascular lumen upon endothelial cell activation. The pivotal physiological role of VWF is to immobilize and activate platelets via binding of the surface glycoprotein GPIb to the A1 domain of VWF^4^, and it may also contribute to coronary artery disease^19^. Notably, under distinct pathological conditions, VWF is able to bind to other cell types, such as sickle cells, leukocytes and tumor cells^4,5,20,21^. VWF even mediates staphylococcus binding to the endothelium^22^. Moreover, many diseases show a coincident increase in VWF plasma levels, eryptosis rates and erythrocyte adhesion to the vascular wall^4^, further supporting our hypothesis.

## Materials and Methods

### Working solutions and reagents

All solutions, recombinant VWF and VWF mutant constructs were prepared as previously described^7,23,24^. All solutions were adjusted to a physiological pH of 7.4 as necessary and filter-sterilized after preparation. HEPES-buffered Ringer’s solution (HBRS) consisted of 125 mmol/l NaCl, 5 mmol/l KCl, 1 mmol/l MgCl_2_, 1 mmol/l CaCl_2_, 5 mmol/l glucose, and 32.2 mmol/l HEPES. Ringer’s solution (300 mOsm), glucose-free solution (300 mOsm) and hypertonic solution (850 mOsm) were prepared as previously described^7,24^. Plasmatic VWF was purchased from Calbiochem, Bad Soden, Germany.

### Erythrocyte preparation

Freshly drawn human whole blood samples were treated with hirudin (Instrumentation Laboratory GmbH, Vienna, Austria) and centrifuged for 7 min at 600 g. The erythrocyte pellet was washed twice using HBRS. Next, 300 µl of washed erythrocytes was transferred into 45 ml of hypertonic solution or glucose-free solution and incubated at 37 °C for 5–6 h or 48 h, respectively. To avoid potential unspecific interactions, the erythrocytes were then washed again and resuspended in Ringer’s solution to a hematocrit of 30% for adhesion experiments.

Measurement of chronic renal failure (CRF) patient blood samples was carried out directly after washing and resuspension, i.e., without intermediate incubation. Identically treated blood samples from healthy donors served as controls.

To test the impact of Annexin V (AV) and ADAMTS13 (A13) on adhesion, freshly isolated erythrocytes were incubated for 30 min with either 10 µg/ml purified recombinant AV (BD Biosciences, Heidelberg, Germany), 5 µg or 25 µg/ml anti-AV antibody (Abcam, Cambridge, UK), 50 µl/ml recombinant ADAMTS13 (Baxter, Vienna, Austria) or 50 IU/ml unfractionated heparin (Biochrom AG, Berlin, Germany) prior to the experiments.

### Flow cytometry measurements

Flow cytometry was performed as previously described^7,24^.

### Microfluidic adhesion experiments and reflection interference contrast microscopy

Microfluidic experiments were performed as previously described^22,25^ using the air pressure-driven microfluidic system BioFlux 200 (Fluxion Biosciences, South San Francisco, CA). Channels of a 48-well BioFlux plate were biofunctionalized for 3 h at 37 °C with either 2% BSA serving as a negative control or plasmatic VWF (100 µg/ml) and rinsed with 0.5% BSA. Channels were perfused for 10 min with different erythrocyte or artificial vesicle preparations at 2 dyne/cm² and 10 dyne/cm², respectively. Reflection interference contrast microscopy (RICM) was used to visualize the interactions of the erythrocytes with the coated channel surfaces.

For RICM visualization, an inverted Microscope Observer Z1 (Zeiss, Jena, Germany) with a Colibri LED illumination system was used at two excitation wavelengths (470 and 555 nm). Images were captured with a monochromatic camera (Axiocam MRm, Zeiss, Jena, Germany). The acquisition time was 180 ms at 200-ms or 350-ms intervals. For image acquisition and processing, we used ZEN software (Zeiss, Jena, Germany). The computerized quantification of erythrocyte-VWF adhesion events was performed using an adaptive Hough transformation model^26^.

### Endothelial cell preparation

Human umbilical vein endothelial cells (HUVECs) were isolated and cultivated as described previously^22,27^. Secretion of VWF was stimulated using 100 nM phorbol myristate acetate (PMA; Sigma-Aldrich, Taufkirchen, Germany) in EGM-2 or 50 µM histamine for 10 min before starting the flow experiments.

Human dermal microvascular endothelial cells (HDMECs) were obtained from PromoCell (Heidelberg, Germany) and cultivated in IBIDI flow channels (IBIDI, Martinsried, Germany) using EGM-2 as described previously^22^. After reaching confluency, 50 µM histamine was used to stimulate WPB exocytosis.

### Immunostaining flow experiments

To quantify the adherent erythrocytes and visualize ULVWFs, immunostaining was carried out and adapted as previously described^5,22^. To quantify the adherent erythrocytes in the VWF-coated channels, FITC-labeled Annexin V-Fluos (Roche Diagnostics, Mannheim, Germany) was used during sample preparation. Immunostaining of the adherent erythrocytes on HUVECs and stimulated HDMECs was carried out as follows: fixation was performed with 4% paraformaldehyde (PFA) for 30 min at 4 °C, followed by washing with 0.5% BSA and blocking with 2% BSA. The primary and secondary antibodies (1:200 in 0.5% BSA) were incubated for 45 min at room temperature. All steps were carried out at 2 dyne/cm² for 5 min in the flow direction used in the initial experiment. Images were captured immediately after a final rinsing step.

The following antibodies were used:

FITC-polyclonal sheep anti-human VWF (GeneTex Inc., Irvine, CA, USA), monoclonal mouse anti-human Band 3 (Sigma-Aldrich, Taufkirchen, Germany), Texas red goat anti-mouse (Invitrogen, Eugene, Oregon, USA).

### Lipid vesicles

Giant unilamellar vesicles were prepared by electroformation as previously described^28^.

### Platelet preparation

Human platelets were prepared as previously described^29^. In brief, citrated blood (Sarstedt, Nuembrecht, Germany) was centrifuged at 120 × g for 15 min at room temperature. Apyrase (75 microunits/ml; Sigma-Aldrich) and 100 nM prostaglandin E1 (Sigma Aldrich) were added to the platelet-rich plasma (PRP) to prevent platelet activation. Platelets were pelleted by centrifugation at 1200 × g for 15 min and washed with washing buffer (36 mM citric acid, 5 mM glucose, 5 mM KCl, 1 mM MgCl_2_, 103 mM NaCl, 2 mM CaCl_2_, 75 microunits/ml apyrase, 100 nM PGE2, 3.5 mg/ml bovine serum albumin, pH 6.5). The washing procedure was repeated twice. Platelets were stored in HEPES/Tyrode’s buffer (137 mM NaCl, 2 mM KCl, 3 mM NaH_2_PO_4_, 1 mM MgCl_2_, 5.5 mM glucose, 5 mM HEPES, 12 mM NaHCO_3_, pH 7.4).

### ELISA

To quantify VWF-AV binding, a modified sandwich ELISA technique using a polyclonal rabbit anti-VWF antibody (Dako, Copenhagen, Denmark) and a polyclonal rabbit peroxidase-labeled anti-human VWF antibody (Dako, Copenhagen, Denmark) were applied. Transparent 96-well plates were coated with either 20 µg/ml purified AV from human placenta (Sigma-Aldrich, Taufkirchen, Germany), 20 µg/ml PS or 2 µg/ml anti-VWF antibody to generate an immobilized substrate. Various concentrations (0.15, 0.5, 1.0 and 1.5 µg/ml) of human plasmatic VWF (Calbiochem, Bad Soden, Germany) were added, and adhesion was quantified colorimetrically. All washing steps were performed with calcium-free buffer (for AV) or detergent-free buffer (for PS).

### Zeta potential measurements

Zeta potentials of erythrocytes were determined after incubation in hypertonic solution (6 h) and glucose-free solution (24 h) and compared with those of erythrocytes that were incubated in Ringer solution as controls. Following incubation, the erythrocytes were centrifuged at 700 × g and resuspended in phosphate buffer with mannitol to obtain an isotonic, low-ionic-strength medium suitable for zeta potential measurements (1.7 mM KH_2_PO_4_, 5.2 mM Na_2_HPO_4_, 300 mM mannitol) by mixed laser Doppler velocimetry and phase analysis light scattering (M3-PALS) using a Malvern Zetasizer NanoZS instrument (Malvern Instruments, Malvern, United Kingdom) fitted with a red laser (λ = 632.8 nm).

CRF patients. A 7.5 ml sample of heparin or hirudin blood was collected from CRF patients (n = 9) and healthy donors (n = 5) and prepared as described above. Informed consent was obtained from all subjects before inclusion. The study was conducted according to the ethical guidelines of our institution and the Helsinki Declaration.

### Mouse experiments

All experimental protocols were conducted in accordance with German law on animal protection and were approved by the Regierungspräsidium in Tübingen. Homozygous VWF^−/−^ mice^30^ (B6.129S2-Vwf ^ tm1Wgr^/J) (Jackson Laboratory/Charles River, Sulzfeld, Germany) were compared with C57Bl6 control mice. Renal ischemia/reperfusion procedures were performed using 4- to 5-month-old male VWF^−/−^ and control mice as described^31^.

### Morphological evaluation of renal histology

For histological analyses, the kidneys were perfused, fixed with 4% PFA and dehydrated before being embedded in paraffin and cut into sections with a thickness of 2 µm, followed by periodic acid Schiff (PAS) and Sirius red staining.

### Localization of VWF after ischemia/reperfusion

Localization of VWF within the injured kidney was investigated by immunofluorescence double staining using confocal laser scanning microscopy (Zeiss LSM 710, Zeiss, Germany). After cutting, antigen retrieval and blocking, formalin-fixed paraffin sections were incubated with polyclonal rabbit anti-human VWF (Dako, Copenhagen, Denmark) and monoclonal rat anti-mouse CD42b (GPIb, emfret Analytics, Eibelstadt, Germany), as well as the secondary antibodies Alexa555-conjugated goat anti-rat (Invitrogen, Eugene, Oregon, USA) and FITC-conjugated goat anti-rabbit (BD Pharmingen, USA), as described previously^6,32^.

### Statistical analyses

STATA12 for Windows was used for the statistical analysis. Data are presented as the means ± s.e.m. Two-sided tests were used throughout the analysis, and the differences were considered statistically significant at *P* < 0.05. Pairwise (univariate) comparisons were performed using Student’s *t* test or the Mann-Whitney *U* test as appropriate.

### Human blood samples

The human erythrocytes used in the experiments were isolated from blood samples from healthy donors or, where indicated, from patients with chronic renal failure. The study was approved by the local ethics committee (Ethics committee II of the Medical Faculty Mannheim, Ruprecht-Karls-University Heidelberg, Mannheim, Germany), and informed consent was obtained from participants prior to inclusion of their samples in the study. All methods were performed in accordance with the relevant guidelines and regulations.

### Data availability

The datasets generated and/or analyzed during the current study are available from the corresponding author upon reasonable request.

## Results

### Eryptotic erythrocytes adhere to VWF and ULVWFs

**Figure 1:**
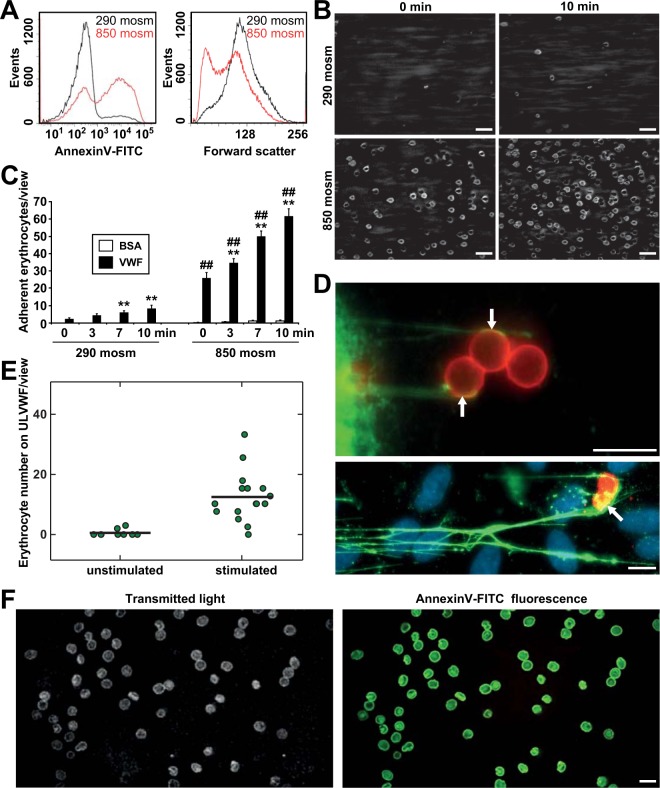
Eryptotic erythrocytes bind to VWF fibers. (**A**) Representative FACS Annexin V (AV) staining and forward scatter of unstressed (black curve) and eryptotic (red curve) erythrocytes after 6 h of incubation at 290 mosm and 850 mosm. **(B)** Representative images of erythrocyte adhesion on a VWF functionalized flow channel visualized by RICM following incubation for 6 h at 290 mosm and 850 mosm after 0 and 10 minutes at a flow rate of 2 dyne/cm^2^. Scale bars correspond to 20 µm. **(C)** Quantification of erythrocyte adhesion on VWF- or BSA-coated flow channels visualized by RICM incubated for 6 h at 290 mosm and 850 mosm after the indicated time at a flow rate of 2 dyne/cm^2^ (n = 4 independent experiments). **(D)** Representative images of adherent eryptotic erythrocytes and erythrocyte clustering on ULVWFs released by human endothelial cells upon stimulation (red: AE-1; green: VWF; blue: DAPI; white arrows indicate ULVWF-erythrocyte binding sites). Scale bars correspond to 10 µm. **(E)** Counting of erythrocytes attached to endothelial-derived VWF strings after 10 minutes of shear flow at 2 dyne/cm^2^. The black bar shows the median number of attached erythrocytes from four independent experiments. **(F)** Comparison of transmitted light and AV-FITC fluorescence demonstrates that only AV-positive erythrocytes bind to VWF-coated surfaces under flow. The scale bar corresponds to 10 µm. **Significance at p < 0.01 compared with the baseline control of the respective group at 0 min, as determined by Student’s t test. ^##^Significance at p < 0.01 between the 850 mosm and corresponding 290 mosm values (Student’s t test).

Human erythrocytes were exposed to hyperosmotic stress or glucose-free solution to generate eryptotic erythrocytes. To mimic within only 6 hours the pathophysiological disturbances resulting from erythrocytic stress that normally develop over a lifetime of approximately 120 days, we had to choose harsh hyperosmotic conditions (850 mosm). Nevertheless, these conditions are physiologically relevant, as they reflect the osmotic situation in the kidney medulla or during inflammation in other organs^33^. This procedure triggered phosphatidylserine (PS) exposure (detected by Annexin-V binding) and cellular shrinkage, which are typical hallmarks of eryptosis (Fig. 1A). Next, perfusion experiments were performed under distinct flow conditions with shear rates of 1.5 to 2 dyne/cm^2^, which are similar to the flow conditions present in microvessels. As shown in Fig. 1, stressed erythrocytes strongly adhered to the VWF-coated surface (Fig. 1B,C,F) and to endothelial-derived ULVWFs (Fig. 1D,E). This adhesion increased over time, whereas significantly fewer unstressed erythrocytes adhered to VWF-coated surfaces. Neither stressed nor unstressed cells bound to surface coatings consisting of BSA (Fig. 1B,C,E,F). Even more significantly attenuated adhesion to VWF functionalized flow channels was observed when the shear rate was increased to 10 dyne/cm^2^, but the optimal interaction probability of this shear dependent binding was shown to occur in an intermediate shear flow of 2 dyne/cm^2^ (Fig. S1). To validate that the interaction between the erythrocytes and VWF is related to stressed erythrocytes in general rather than a specific response to hyperosmotic stress, we also analyzed the interaction using erythrocytes subjected to glucose depletion, which is a trigger of eryptosis that is mechanistically unrelated to hyperosmotic stress. Glucose depletion resulted in the same effect as hyperosmotic stress with even similar extents, suggesting an effect that is independent of the type of stress as long as it induces eryptosis (Fig. S2A,B). To study the adhesion of eryptotic erythrocytes to human endothelial cells (ECs), we coated the microfluidic channels with human umbilical vein endothelial cells (HUVECs). Upon EC activation, stressed erythrocytes bound to EC-released ULVWFs as single cells or cell clusters (Fig. 1D,E and Fig. S3A–C). The dynamics of this binding are depicted in Supplementary Movies 1–4 (Movie 1: control erythrocytes on control ECs, Movie 2: control erythrocytes on activated ECs, Movie 3: stressed erythrocytes on control ECs, and Movie 4: stressed erythrocytes on activated ECs) and reveal transient or firm adhesion that decelerates the resulting erythrocyte flow. The distinct characteristics of both transient interactions and adhesion events of stressed erythrocytes on activated ECs are illustrated by a z-axis-oversubscribed three-dimensional RICM reconstruction movie with the focal plane at the microfluidic luminal EC surface (Supplementary Movie 5).

Moreover, our data showed that EC-derived VWF facilitates erythrocyte adherence to ECs: No erythrocyte adhesion was detected on non-stimulated ECs, as they lacked ULVWF formation (Fig. 1E). Consistently, in a cell suspension containing stressed and unstressed erythrocytes, Annexin V (AV) staining revealed that all VWF-adherent erythrocytes were PS-positive, reflecting eryptotic erythrocytes (Fig. 1F). To further consolidate the more pronounced binding of stressed erythrocytes to VWF, we performed additional flow cytometry measurements. Fig. S4 shows that almost exclusively eryptotic, but not vital, controlled erythrocytes bound to eGFP-labeled VWF.

### VWF binds to phosphatidylserine and Annexin V on the erythrocyte surface

**Figure 2:**
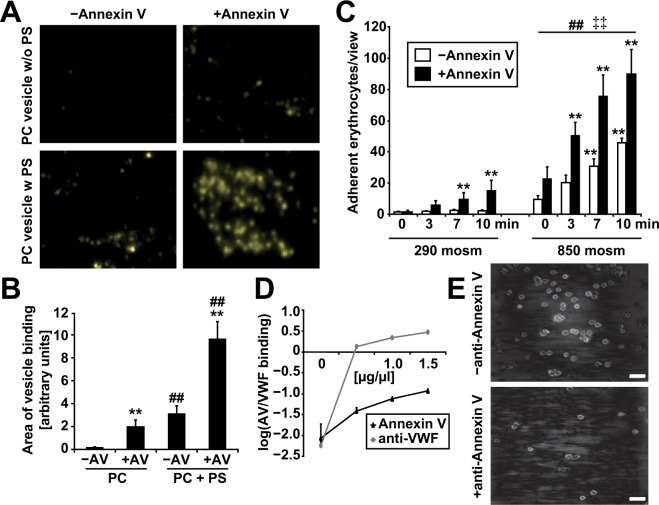
VWF binds to PS and AV on erythrocytes. (**A)** and **(B)**, Binding of phospholipid (PC: phosphatidylcholine; PS: phosphatidylserine) vesicles to the VWF-coated flow channel with and without AV addition after 0 and 10 min of flow; **(A)** original images and **(B)** quantification (n = 3). **Significance at p < 0.01 compared with the baseline control without Annexin V, as determined by the two-sided Student’s t test. ^##^Significance at p < 0.01, PC + PS vesicle binding compared with PC-only vesicle binding (two-sided Student’s t test). **(C)** Quantification of erythrocyte adherence in a VWF-coated flow channel visualized by RICM following incubation for 6 h at the indicated osmolarity in the absence and presence of AV (n = 4 independent experiments). **Significance at p < 0.01 compared with the baseline control of the respective group at 0 min, as determined by the two-sided Student’s t test. ^##^Significance at p < 0.01 of 850 mosm values compared with the corresponding 290 mosm values (two-sided Student’s t test). ^‡‡^Significance at p < 0.01 of erythrocyte binding in the absence of AV compared with erythrocyte binding with AV present (two-sided Student’s t test). **(D)** Binding of AV and VWF *in vitro* as measured by ELISA compared with VWF antibody as a positive control (n = 4). (**E**) Representative original image of erythrocyte adherence in a VWF-coated flow channel visualized by RICM following incubation for 6 h in 850 mosm solution in the absence and presence of anti-AV. Scale bars correspond to 20 µm.

**Figure 3:**
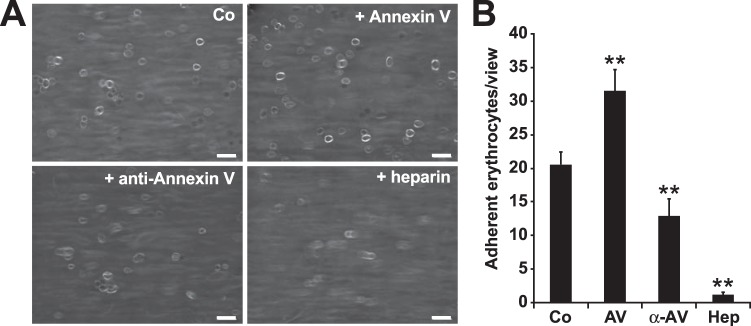
Eryptotic erythrocyte binding to endothelial-derived ULVWFs controlled by Annexin V. (**A)** Representative RICM images and **(B)** Hough transformation quantification of hypertonic-stimulated (850 mosm) erythrocyte adherence (Co) supplemented with Annexin V (AV), an anti-Annexin V antibody (α-AV) or heparin (Hep) as indicated after 10 minutes of flow at 1.5 dyne/cm² (n = 4). Scale bars correspond to 20 µm. **p < 0.01, as determined by the two-sided Student’s t test.

Numerous adhesion molecules have been shown to interact with VWF and promote binding, such as collagen, integrins, and P-selectin^30,34^. Most of these molecules are, however, not expressed in mature erythrocytes. Since PS exposure evidently alters the surface of eryptotic erythrocytes, we hypothesized that PS exposure may be responsible for the enhanced VWF binding. To find evidence for a direct VWF-PS interaction, VWF was subjected to different lipid vesicle model systems. Vesicles containing only phosphatidylcholine (PC) represent the membrane of unstressed erythrocytes, whereas the addition of 50% PS yields a model of the eryptotic erythrocyte membrane. Under flow conditions, these PS-containing vesicles engaged in significant binding to VWF, whereas pure PC vesicles did not (Fig. 2A). Surprisingly, the addition of AV, which is supposed to saturate free PS binding sites and thereby decrease erythrocyte binding, increased the binding of the PS-containing vesicles to VWF (Fig. 2A,B). Likewise, we found that the addition of AV to PS-exposing erythrocytes also enhanced their adhesion to VWF (Fig. 2C). These data suggest that cell surface-bound AV, but not PS alone, promotes binding to VWF. Because the binding of erythrocytes to VWF may be at least partially triggered by electrostatic interactions, we further tested whether the exposure of PS could affect the negative surface charge of erythrocytes. The zeta potential of stressed and vital erythrocytes was comparable (Fig. S5A,B), indicating that the exposure of PS did not significantly alter the zeta potential. Therefore, we hypothesize that AV binds not only to PS but also to VWF and thereby anchors the PS-exposing membrane to VWF or the vessel wall. AV can be externalized to the cell surface of different cell types as a reaction to cellular stress or during the course of apoptosis^35,36^. Because AV expression has not yet been described in erythrocytes, we performed additional experiments to verify AV expression in erythrocytes. AV was expressed in vital, mature erythrocytes (Fig. S6A), but it was almost absent on the cell surface. In contrast, stressed (hypertonic and glucose-free) erythrocytes showed a significant increase in AV externalization to their cell surface (Fig. S46B,C). To confirm the AV-VWF binding properties, we established an ELISA assay. Figure 2D shows the existence of concentration-dependent VWF-AV binding using recombinant AV and VWF. Consistently, the addition of an antibody against AV efficiently inhibited the adhesion of stressed erythrocytes to a VWF-coated surface (Fig. 2E). Focusing on adhesion events of eryptotic erythrocytes on EC-derived ULVWFs, we next quantified the functional impact of AV. As shown in Fig. 3A,B, the addition of AV to the samples prior to microfluidic examination led to an enhanced number of adhesion events, whereas the addition of an anti-AV antibody decreased erythrocyte-ULVWF interactions (for the dynamics of the depicted set of still images in Fig. 3A, see also Supplementary Movie 6).

Taken together, these data show that eryptotic erythrocytes express and externalize PS and AV, which mediate the binding to VWF.

### VWF binds to erythrocytes via its A1 domain, which can be blocked by ADAMTS13 or heparin

**Figure 4:**
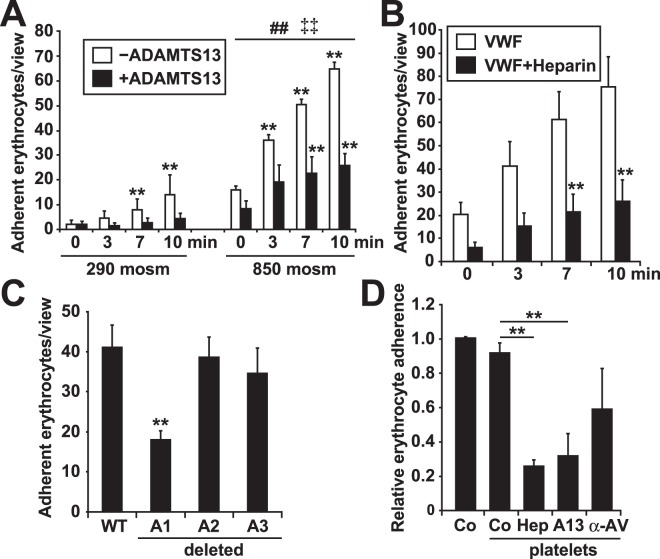
Mechanism of VWF-erythrocyte binding. (**A)** Quantification of erythrocyte adherence in a VWF-coated flow channel visualized by RICM following incubation for 6 h at the indicated osmolarity in the absence and presence of ADAMTS13 (n = 4). **Significance at p < 0.01 compared with the baseline control of the respective group at 0 min, as determined by the two-sided Student’s t test. ^##^Significance at p < 0.01 of 850 mosm values compared with the corresponding 290 mosm values (two-sided Student’s t test). ^‡‡^Significance at p < 0.01 of erythrocyte binding in the absence of ADAMTS13 compared with erythrocyte binding with ADAMTS13 present (two-sided Student’s t test). **(B)** Quantification of erythrocyte adherence in a VWF-coated flow channel visualized by RICM following incubation for 6 h in hypertonic solution (850 mosm) in the absence and presence of heparin (n = 3). **Significance at p < 0.01 compared with the baseline control of the respective group at 0 min, as determined by the two-sided Student’s t test. All values of erythrocyte adherence in the presence of heparin are significantly different (p < 0.01) from the control. **(C)** Quantification of erythrocyte adherence after 10 minutes of flow in a flow channel coated with either wild-type VWF or VWF mutants with deletions in the A1, A2 or A3 domains as indicated, visualized by RICM following incubation for 6 h in hypertonic solution (850 mosm) (n = 4). **Significance at p < 0.01 compared with wild-type VWF (two-sided Student’s t test). **(D)** Quantification of erythrocyte adherence in a VWF-coated flow channel after 10 minutes of flow in the presence or absence of platelets and the indicated additional inhibitors of adherence (heparin (Hep), ADAMTS13 (A13) anti-Annexin V antibody (α-AV)) (n = 4). **Significance at p < 0.01 compared with the control without platelets (two-sided Student’s t test).

To gain additional insight into molecular mechanisms and to develop possible prognostic or therapeutic approaches, we tested the effect of different factors that are known to inhibit VWF binding capacity. First, we investigated the impact of the VWF-specific protease ADAMTS13 on VWF-erythrocyte adhesion. The addition of ADAMTS13 significantly reduced the adhesion of both stressed and unstressed erythrocytes to VWF-coated surfaces under flow conditions (Fig. 4A). Second, VWF is known to bind to numerous substrates via its positively charged binding sites^37^. Previously, we were able to show that negatively charged macromolecules, such as heparin or DNA, compete with other VWF binding partners^25^. Similar to ADAMTS13, the addition of heparin significantly attenuated the adhesion of erythrocytes to VWF (Fig. 4B). These findings indicate that shear-activated VWF, PS/AV and VWF A1 domains are mainly involved in the process of VWF-erythrocyte adhesion. To confirm the VWF A1 domain as being mainly responsible for erythrocyte binding, we tested the adhesion ability of recombinant VWF molecules lacking different A domains. Figure 4C shows that the erythrocyte binding capacity was decreased by 50% in VWF lacking its A1 domain compared with that in the wild-type VWF. Thus, we could rule out the involvement of the A2 and A3 domains because we could not show any decrease in adhesion in assays involving recombinant VWF proteins lacking these domains (Fig. 4C). This result indicates that the A1 domain of VWF is one main binding partner responsible for VWF-erythrocyte adhesion. It is known that the A1 domain is the main binding site for GPIb expressed on platelets. Therefore, we added platelets as a competing binding partner. Upon shear stress at 2 dyne/cm², the binding of eryptotic erythrocytes to VWF did not significantly change in the presence of platelets, whereas additional coincubation with heparin, ADAMTS13 or an anti-AV antibody strongly attenuated erythrocyte adhesion (Fig. 4D).

### Eryptotic erythrocytes obtained from chronic renal failure patients immobilize on a VWF functionalized surface under flow conditions

**Figure 5:**
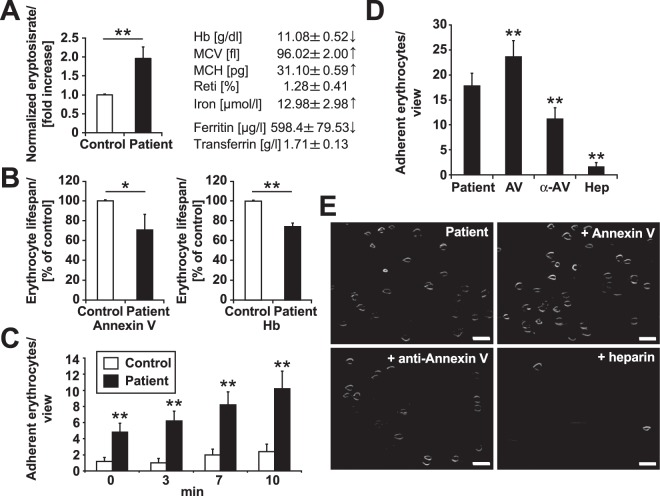
CRF patient erythrocytes show both enhanced eryptosis and VWF binding. (**A)** Percentage of AV-positive erythrocytes in healthy control individuals (left bar; n = 5) and CRF patients (right bar; n = 5) measured by FACS from freshly isolated erythrocytes. The right table shows CRF patient blood parameters regarding anemia. The arrows indicate elevated or decreased values compared with normal values. **(B)** Calculation of erythrocyte lifespan in healthy control individuals (left bar; n = 5) and in CRF patients (right bar; n = 5) based on either AV-positivity (left graph) or hemoglobin content (right graph). For the first calculation, the formula (100%*eryptosis rate(patient)/eryptosis rate(control) = %lifespan of patient erythrocytes) was used; for the second calculation, the formula (100% * hemoglobin(patient)/hemoglobin(control) = %lifespan of patient erythrocytes) was used. **(C)** Quantification of spontaneous adherence of erythrocytes from healthy control donors (white bars) and CRF patients (black bars) in a VWF-coated flow channel visualized by RICM (n = 5). **(D)** Quantification and **(E)** representative RICM images of spontaneous erythrocyte adherence of CRF patient samples that were untreated (control) or supplemented with Annexin V (AV), an anti-Annexin V antibody (α-AV) or heparin (Hep), as indicated, after 10 minutes of flow at 1.5 dyne/cm² (n = 4). Scale bars correspond to 20 µm. *p < 0.05; **p < 0.01 (two-sided Student’s t test).

To confirm the clinical relevance of our *in vitro* data, we analyzed blood samples from patients suffering from kidney failure. The kidney is highly sensitive to disorders related to microangiopathy or capillary occlusion. In addition, acute renal failure leads to the formation of compounds that are known to trigger eryptosis, such as prostaglandin E2, ceramides, and several uremic toxins^14^. Therefore, we correlated the numbers of circulating eryptotic erythrocytes in patients suffering from CRF with the degree of erythrocyte-VWF adhesion. First, we found that the number of PS-exposing erythrocytes detected in dialysis patients was almost twice as high as that in healthy individuals under resting conditions (Fig. 5A). Despite sufficient erythropoietin substitution, dialysis patients commonly experience anemia^14^. On the basis of either PS exposure or hemoglobin values, our calculations revealed a more than 20% lower lifespan of erythrocytes from CRF patients than healthy donors (Fig. 5B). In agreement with these results, we found a greater number of erythrocytes adhering to VWF-coated channels in CRF patient samples than healthy control erythrocytes, underlining the positive correlation of erythrocyte PS exposure and VWF adherence in clinically relevant settings (Fig. 5C). Moreover, this enhancement of erythrocyte adhesion to VWF observed in CRF patient samples could be directly modified by AV. The addition of AV to the samples prior to microfluidic examination led to a further increase in erythrocyte-VWF interactions under flow conditions, whereas an anti-AV antibody showed a decreased effect (Fig. 5D,E). Interestingly, heparin prevented erythrocyte adhesion to VWF in CRF patient samples comparable to that observed in samples of healthy individuals. These data confirm the impact of AV on eryptotic erythrocyte-VWF binding and the function of heparin as an inhibitor.

### VWF deficiency prevents EC erythrocyte adhesion and kidney damage upon ischemia reperfusion injury in mice

**Figure 6:**
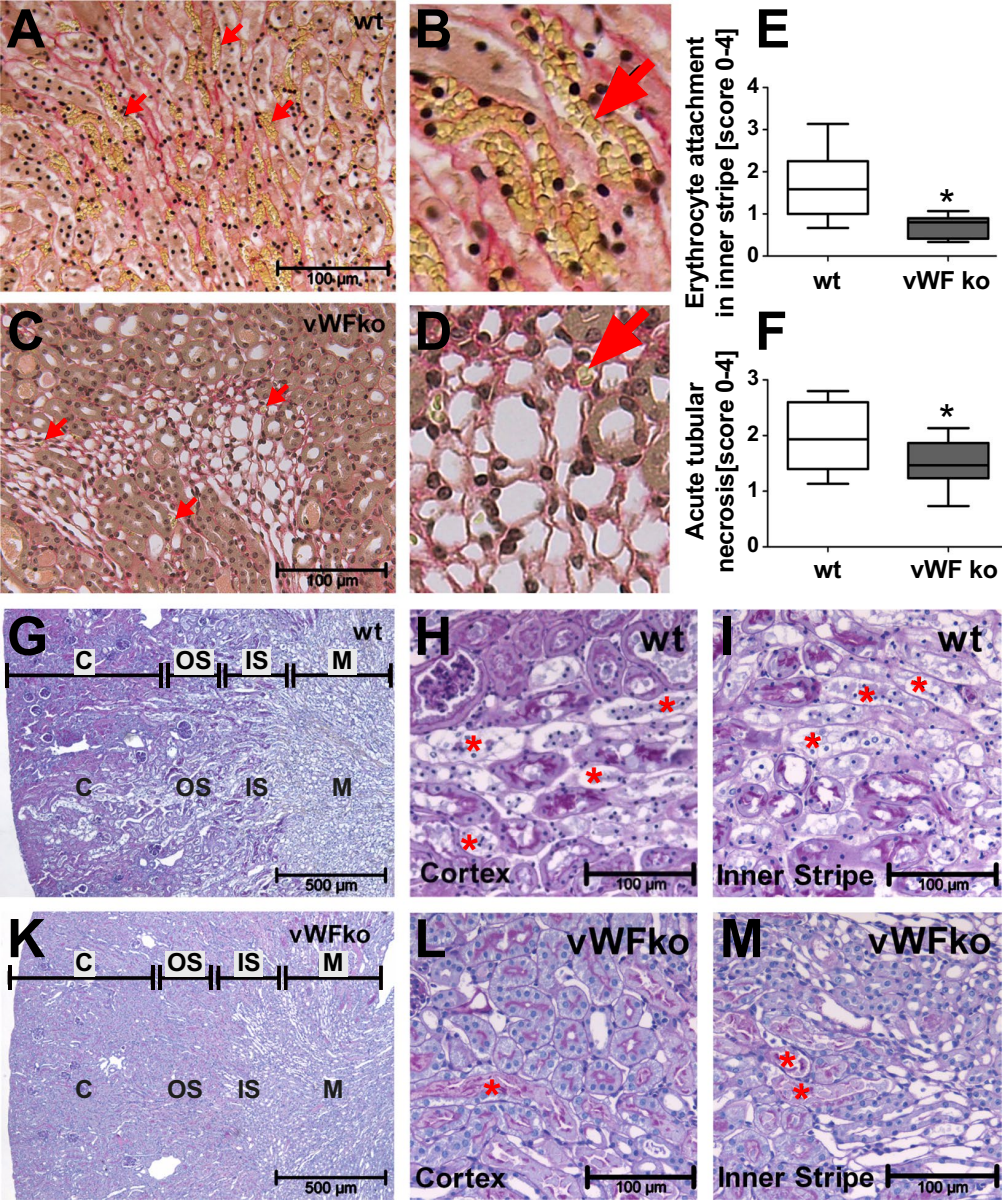
VWF^−/−^ mice show diminished erythrocyte accumulation and kidney damage after ischemia reperfusion injury (IRI) compared with wild-type (WT) mice. Erythrocyte attachment (red arrows) in the inner stripe of WT (**A,B**, n = 11; representative image) and VWF-deficient mice (**C,D**, n = 9; representative image) monitored after IRI using the Sirius red stain (erythrocytes indicated in yellow). (**E**) Quantification of erythrocyte attachment according to the scoring system depicted in Suppl. Table 1. (**F**) Tubular necrosis according to the scoring system depicted in Suppl. Table 2. (**E,F**) *p < 0.05, as determined by the Mann-Whitney u-test. Representative PAS-stained sections showing severely damaged tubuli in different compartments of WT kidneys (**G**) and only mild damage in VWF-deficient mice (**K**, magnification 40× each). Higher magnification (200×) showing distinct tubular necrosis (examples marked in red *) in an IRI-damaged WT cortex (**H**) as well as in the inner stripe (**I**). VWF-deficient kidneys showing less damage and a more preserved morphology in the cortex (**L**) and inner stripe (**M**) after IRI. C = cortex; OS = outer stripe; IS = inner stripe; M = medulla.

Next, we tested the relevance of our findings in an *in vivo* model of renal ischemia/reperfusion injury (IRI), a condition associated with increased eryptosis. IRI stress directly damages the renal and vascular tissue by oxidative stress within minutes^38^. The erythrocytes become eryptotic due to oxidative stress as well as secondary toxic stress caused by the activated endothelial cells during the first 24 h. One day after IRI model induction, numerous erythrocytes adhered to the peritubular capillaries of the IS in wild-type kidneys (Fig. 6A,B, arrows). In contrast, the erythrocytes of the VWF^−/−^ mice showed hardly any adhesion in this area of the kidney after IRI (Fig. 6C,D, arrows). This reduction in erythrocyte binding mitigated the renal damage after the induction of IRI in VWF^−/−^ mice compared with that in wild-type mice, as reflected by a reduced rate of acute tubular necrosis, tubular dilatation and atrophy in VWF^−/−^ mice (Fig. 6E,F, Tables S1 and 2). Tubular necrosis was pronounced in wild-type kidneys in the IS and outer stripe (OS), as well as in the cortex (C) (Fig. 6G,H,I, asterisks). In the VWF^−/−^ mice, tubular necrosis was reduced in the IS and was almost absent in the cortex (Fig. 6K–M, asterisks).

### Intraluminal VWF mediates erythrocyte adherence *in vivo* upon renal damage

**Figure 7:**
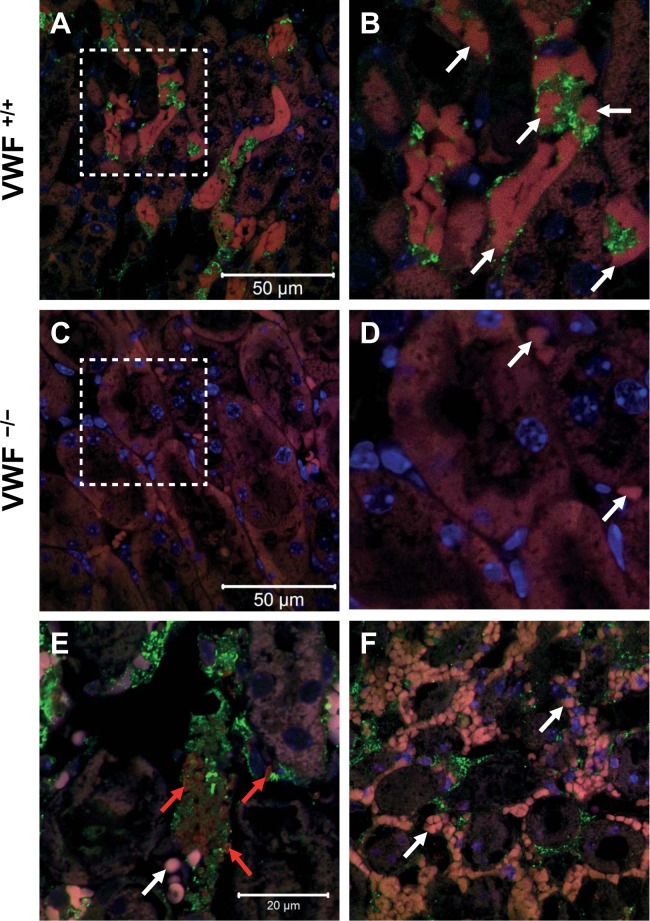
Intraluminal VWF specifically binds erythrocytes *in vivo* upon renal damage. (**A–F)** Representative immunostaining for VWF in kidneys of 11 wild-type **(A**,**B**,**E**,**F)** and 9 VWF^−/−^ mice **(C**,**D)** after IRI at two different magnifications each **(A–D)**. In wild-type mice **(A**,**B)**, but not in VWF^−/−^ mice **(C**,**D)**, a large number of erythrocytes (marked with white arrows in B and D) and VWF (green) are visible. 6B and D represent higher magnifications of 6 A and C, respectively. To visualize platelets, we performed staining using anti-GPIb antibodies **(E**,**F)**. In 6E, platelet clusters (marked with red arrows) are observed colocalized with VWF (green). Erythrocytes (pale pink, marked with white arrows) are barely visible (E). In contrast, in areas of high erythrocyte aggregation **(F)**, we were unable to identify platelets (no red staining, but erythrocytes are marked with white arrows as pale pink autofluorescence). Blue DAPI staining marks cellular nuclei. Intraluminal erythrocytes show pale pink to red autofluorescence.

To specify the erythrocyte adherence to the endothelium in the IRI mouse model, we performed several immunofluorescence staining assays using *ex vivo* kidney tissue sections after IRI in wild-type and VWF^−/−^ mice. Since our *in vitro* data (Fig. 1) and *in vivo* analysis (Fig. 6) indicated that VWF was mainly responsible for erythrocyte adhesion to the vessel wall, we performed VWF staining using anti-VWF antibodies. The erythrocytes were not stained specifically, as they could be easily detected due to their high autofluorescence. In wild-type mice, large amounts of intraluminal VWF attached to erythrocytes (autofluorescence in red, arrows) were visible in renal capillaries (Fig. 7A,B). The pattern and morphology of VWF staining suggested the presence of high-molecular-weight VWF multimers resembling ULVWFs. These ULVWFs bound to a large number of deformed and eryptotic erythrocytes, leading to almost complete occlusion of the capillary lumen (Fig. 7A,B). In contrast to wild-type mice, almost no erythrocytes (autofluorescence in red, arrows) could be detected within the renal capillaries in VWF^−/−^ mice (Fig. 7C,D). The obvious occlusion of renal capillaries in wild-type mice prevented reperfusion-mediated reoxygenation and therefore increased tubular cell damage. These phenomena explain the greatly reduced tubular damage in VWF^−/−^ compared with that in wild-type mice (Figs 6 and 7A–D). To study possible interference between erythrocyte and platelet binding to ULVWFs *in vivo*, we further stained wild-type mouse kidneys for VWF and GPIb (expressed by platelets). As shown in Fig. 7E, we could detect areas of VWF accumulation bound to a large number of platelets (stained in red, red arrows). However, these areas were virtually free of erythrocytes (Fig. 7E, white arrow, visible as a pale pink autofluorescence signal). However, most of the renal capillaries showed significant erythrocyte (visible as a pale pink autofluorescence signal) binding in the lumen (white arrows in 7 F denote erythrocytes only). Although we detected VWF networks in these vessel sections, we were not able to detect platelets attached to erythrocytes, in contrast to a VWF network in the vessel lumen (Fig. 7F). This finding clearly illustrates that the adherence of eryptotic erythrocytes to the endothelial cells was independent of the presence or mediation of platelets (Fig. 7E,F). These *in vivo* results corroborate our *in vitro* data showing that intraluminal VWF mediates non-platelet-dependent erythrocyte binding to ECs and subsequent vascular occlusion as well as renal tubular damage.

To further validate a putative interaction of stressed erythrocytes with the endothelium *in vivo*, we performed intravital microscopy in the dorsal skinfold chamber (DSC), which allows continuous *in vivo* observation of *ex vivo* fluorescently labeled and transfused cells in the cutaneous microcirculation^39^. Healthy mice equipped with a DSC received either unstressed erythrocytes incubated in Ringer solution or hypertonic stressed erythrocytes. As shown in Supplementary Figure 7, adherent stressed erythrocytes could be localized in cutaneous blood vessels (red arrows), whereas unstressed erythrocytes were not detectable.

## Discussion

**Figure 8:**
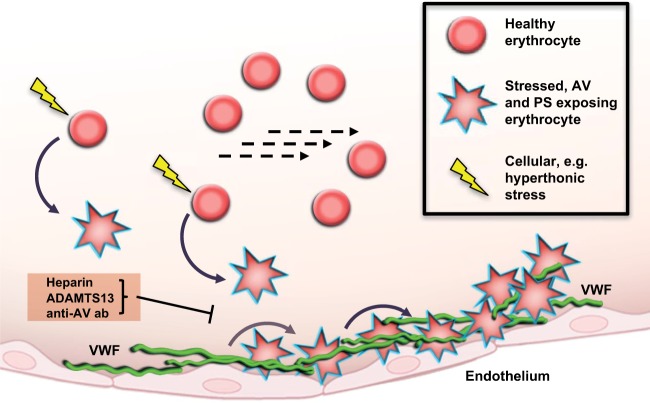
Proposed mechanism of erythrocyte contribution to vascular occlusion via VWF binding. Upon cellular stress, erythrocytes show deformation, shrinkage and exposition of phosphatidylserine (PS) as well as Annexin V (AV). Intravascular VWF of activated endothelium binds surface AV of these stressed erythrocytes, whereas unstressed, vital erythrocytes do not adhere. Although this binding is mainly reversible, it can still lead to an impairment of dynamic blood flow and vascular occlusion. This binding can be blocked by anti-AV antibody, heparin and ADAMTS13.

Our results comprehensively illustrate the direct interaction between erythrocytes and VWF using *in vitro* and *in vivo* models. We characterized the binding process and elucidated the underlying molecular mechanism (Fig. 8). Thus, our study contributes to the identification of a relevant source of vascular damage and microvascular occlusion. Finally, we propose the use of heparin and ADAMTS13 as a therapeutic approach to treat microvascular occlusion (Fig. 8).

Previous studies postulated a possible interaction between erythrocytes and VWF only for deformed sickle cells or artificial cell stress^20,40^. In sickle cell anemia, binding to VWF is, in contrast to our results, attributed to a genetic hemoglobin disorder, which manifests as deformed erythrocytes with reduced plasticity. Bridges *et al*. showed that plasmodium-infected erythrocytes bind to VWF adherent platelets but not to VWF fibers directly^41^. In 2015, erythrocytes were found in ULVWFs in a microfluidic system similar to ours, but the role of erythrocytes in microangiopathy and the binding mechanism of erythrocytes were not elucidated^42^. Our data are consistent with a recently published manuscript by Smeets *et al*., who showed that stressed erythrocytes bind to ULVWFs *in vitro* but did not provide mechanistic data or *in vivo* relevance^40^.

Our presented data therefore expand the sparse existing evidence mentioned above regarding the contribution of erythrocytes to vascular damage and occlusion in two regards. First, we used our microfluidic systems in combination with RICM and fluorescence microscopy to visualize the adherence of erythrocytes to VWF in a dynamic flow system. This procedure allowed us to characterize the degree of cellular adherence under physiological conditions as well as the impact of potential inhibitors or enhancers on this binding process. Second, we identified the A1 domain of VWF and AV, as well as PS on the erythrocyte surface, as the most important binding partners mediating a platelet-independent interaction.

As Chen and coworkers already described for interactions between VWF A1-domain interactions and corpuscular elements under flow conditions^43^, this interaction occurs rapidly and is mainly reversible under physiological flow conditions. However, despite the mainly reversible characteristics of this adherence, deceleration and thus impairment of dynamic blood flow can still occur. Our results show that adherence to VWF is limited to deformed or eryptotic erythrocytes, whereas vital erythrocytes show no binding. In combination with the overwhelming erythrocyte percentage of approximately 95% among corpuscular blood elements, including up to 6% of eryptotic erythrocytes, this effect can account for an instantaneous and physiologically relevant microvascular occlusion. The binding capacity of VWF mainly depends on shear flow, as shown for platelets, bacteria and leukocyte adhesion^5,22,44^. Therefore, in our experiments, we mimicked the physiological shear rates present in different vascular systems and showed that distinct shear rates are needed to activate VWF and erythrocyte adhesion. These shear rates reflect the situation in capillaries and small venules. The relevance of this effect on blood vessels with a narrow lumen is underlined by the fact that the size of single adherent erythrocytes would suffice to occlude small capillaries or substantially increase vessel resistance.

Based on our *in vitro* data, we were able to determine the molecular components of erythrocyte adherence to VWF, namely, the A1 domain of VWF and AV and PS lipids on the surface of eryptotic erythrocytes. Shear stress is known to elongate and activate VWF, a prerequisite for A1 domain exposure and binding capacity^44^. Therefore, microvessels with high shear flow conditions are particularly susceptible to this VWF-erythrocyte interaction. The physiological antagonist of shear flow-induced VWF fiber formation and VWF binding properties is ADAMTS13^45^.

The protease ADAMTS13 regulates the concentration of active VWF oligo- and polymers via cleavage of the A2 domain within VWF^46^. ADAMTS13 activity is decreased in many diseases associated with vascular inflammation or occlusion, such as cancer, TTP, sepsis, malaria and HUS^5,47,48^. All of these conditions are also linked to increased eryptosis^14^. The lack of ADAMTS13 activity or expression leads to excessive shear-induced VWF activation, which also contributes to vascular damage and inflammation^46^. In addition, VWF concentrations appear to increase and function as a cofactor in vasoocclusive diseases such as myocardial infarction or cerebrovascular disease^19,49,50^. Therefore, in diseases that are associated with ADAMTS13 deficiency or excessive VWF release, microangiopathy by the VWF-erythrocyte interaction is supported by the combination of pathological alterations in both erythrocytes via increased eryptosis and VWF via increased release and the lack of VWF degradation. Blocking either side of this cooperative binding causes a dramatic reduction of adherence, as shown herein by the addition of anti-AV antibody, heparin or recombinant ADAMTS13.

Finally, the *in vivo* data from our mouse and human studies support these *in vitro* findings. We confirmed our data by *in vivo* imaging of stressed erythrocyte binding to the vessel wall using a dorsal skin fold chamber. We show that VWF-erythrocyte binding substantially contributes to microvascular damage during acute or chronic renal failure. Loss of renal function often occurs in diseases that are associated with increased eryptosis, such as diabetes, sickle cell disease or HUS^12,24^. Patients suffering from chronic renal failure simultaneously show increased eryptosis rates and increased adherence of their erythrocytes to VWF (Fig. 5). In our IRI mouse model, acute renal failure correlates with erythrocyte adherence to the endothelium of renal capillaries in wild-type animals. We could show the colocalization of intravascular VWF and adherent deformed erythrocytes in renal capillaries. Mice lacking VWF show markedly decreased erythrocyte adhesion in the glomerular capillaries and tubular vessels of IRI-damaged tissue. This finding also illustrates the relevance of VWF-erythrocyte binding as a cause of microvascular occlusion *in vivo* under pathological conditions. In this context, it was recently shown that high sodium-triggered VWF release contributes to vascular occlusion in kidney capillaries^51^. In addition, HUS symptoms in mice are by far more pronounced in the absence of ADAMTS13, validating the clinical relevance of erythrocyte-VWF binding^13,52^. Therefore, inhibition of VWF activity and subsequent erythrocyte binding to VWF by factors such as ADAMTS13 or especially heparins may be an interesting therapeutic option in the treatment of microangiopathies in the near future. In particular, heparin treatment improves the symptoms and clinical course of ulcus cruris diabeticorum or livedoid vasculopathy, both of which are suspected to be related to microvessel occlusion^53,54^. Although the epidemiological relevance of microangiopathy is substantial, existing therapeutic or preventive therapies have thus far been rather ineffective^55^. In this study, we provide a mechanistic explanation for this so far only descriptive clinical effect. Our results suggest that efficient therapy or prevention of microangiopathy through inhibition of the erythrocyte-VWF interaction may decrease the rate of microangiopathic complications in various diseases, such as diabetes, HUS and sickle cell disease. Finally, our data show that heparin or ADAMTS13 mitigate this interaction and may, therefore, be therapeutic options for these microangiopathic disorders. In addition, an anti-VWF antibody, caplacizumab, has already demonstrated promising clinical results in the treatment of thrombotic thrombocytopenic purpura and may also prevent microangiopathy in diseases associated with increased eryptosis^56^. Although our results demonstrate a new mechanism of microcirculation breakdown and describe a promising avenue for the use of VWF-inhibiting agents such as heparin as a therapeutic option for microangiopathy, they must of course be qualified by the derivation of the CRF patient data from *in vitro* results and be used to provide correlations rather than causality. In addition, the mouse *in vivo* model corroborates findings for acute renal failure. Causality in the connection between eryptosis and erythrocyte-VWF adherence could, however, be shown by our *in vitro* results using isolated erythrocytes from healthy donors and artificial eryptosis stimuli, but not in primary patient material.

In summary, increased erythrocyte stress and eryptosis are linked to the pathology of several diseases, such as sickle cell disease, sepsis, chronic kidney disease, hemolytic uremic syndrome, hepatic failure, Wilson’s disease and diabetes^7,14^. All of these conditions show, at least in late stages, microangiopathic vascular damage associated with consecutive loss of organ function. Given the high epidemiological and clinical importance of the abovementioned diseases, our results regarding the genesis and reversibility of erythrocyte binding to endothelial cells via VWF provide new insights into the pathophysiology of microangiopathy and suggest new therapeutic options such as ADAMTS13 or heparin.

## Electronic supplementary material

Supplementary Material

Supplementary Video 1

Supplementary Video 2

Supplementary Video 3

Supplementary Video 4

Supplementary Video 5

Supplementary Video 6
