# Supplementary material for: Cellular stress induces erythrocyte assembly on intravascular von Willebrand factor strings and promotes microangiopathy
Source: Sci Rep. 2018 Jul 19;8:10945. doi: 10.1038/s41598-018-28961-2 (PMC6053440; doi:10.1038/s41598-018-28961-2)
Supplement: Supplementary file 1 — Supplementary Material [file 41598_2018_28961_MOESM1_ESM.pdf]

Cellular stress induces erythrocyte assembly on intravascular von Willebrand factor strings and promotes microangiopathy

Jan P. Nicolay, Verena Thorn, Christoph Daniel, Kerstin Amann, Balasaheb Siraskar, Florian Lang, Carina Hillgruber, Tobias Goerge, Stefan Hoffmann, Christian Gorzelanny, Volker Huck, Christian Mess, Tobias Obser, Reinhard Schneppenheim, Ingrid Fleming, Matthias F. Schneider, and Stefan W. Schneider

Supplementary Materials

Supplementary methods:

Working solutions and reagents. All solutions were adjusted to a physiological pH of 7.4 as necessary and were filter sterilized after preparation. HEPES-buffered Ringer's solution (HBRS) consisted of 125 mmol/l NaCl, 5 mmol/l KCl, 1 mmol/l $MgCl_2$, 1 mmol/l $CaCl_2$, 5 mmol/l glucose, and 32.2 mmol/l HEPES. Ringer's solution (300 mOsm), glucose-free solution (300 mOsm) and hypertonic solution (850 mOsm) were prepared as previously described [1, 2]. Plasmatic VWF was purchased from Calbiochem, Bad Soden, Germany. Recombinant VWF and the VWF mutant constructs were prepared as previously described [3].

Human blood samples. The human erythrocytes for performing the experiments were isolated from blood samples of healthy donors or, where indicated, from patients with chronic renal failure. Written informed consent was received from participants prior to inclusion of their samples in the study.

Erythrocyte preparation. Freshly drawn human whole blood samples were treated with hirudin (Instrumentation Laboratory GmbH, Vienna, Austria) and centrifuged for 7 min at 600 g. The erythrocyte pellet was washed twice using HBRS. 300 µl of washed erythrocytes were transferred into 45 ml of hyperosmolar solution or glucose-free solution and incubated at 37°C for 5-6 h or 48 h, respectively. To avoid potential unspecific interactions the erythrocytes were then washed again and resuspended in Ringer's solution to a hematocrit of 30 % for adhesion experiments.
Measurement of the chronic renal failure (CRF) patients' blood samples was carried out directly after washing and resuspension, i.e. without intermediate incubation. Identically treated blood samples from healthy donors served as controls.
To test the impact of AV and ADAMTS13 on adhesion, freshly isolated erythrocytes were incubated for 30 min with either 10 µg/ml purified recombinant AV (BD Biosciences, Heidelberg, Germany), 5 µg or 25 µg/ml anti-AV antibody (Abcam, Cambridge, UK), 50 µl/ml recombinant ADAMTS13 (Baxter, Vienna, Austria) or 50 IU/ml unfractionated heparin (Biochrom AG, Berlin, Germany) prior to the experiments.

FACS measurements. To determine phosphatidylserine (PS) exposure, the cells were washed in AV binding buffer containing 125 mmol/l NaCl, 10 mmol/l HEPES (pH 7.4) and 5 mmol/l $CaCl_2$. The erythrocytes were stained with Annexin V-Fluos (1:50; Roche Diagnostics, Mannheim, Germany) for 15 min or incubated with eGFP-labeled VWF (provided by Prof. Schneppenheim, Hamburg, Germany) for 30 min. Then, the samples were washed, diluted 1:5 and measured by flow cytometry using a FACS-Canto II (BD Bioscience, Heidelberg, Germany). AV and VWF fluorescence intensities were measured in the FITC channel. In addition, the forward scatter was recorded to determine the cell volume. Data were analyzed with BD FACSDIVA software (BD Bioscience, Heidelberg, Germany) and GraphPad Prism (GraphPad Software, San Diego, USA).

Microfluidic adhesion experiments. Microfluidic experiments were performed using the BioFlux 200 air pressure-driven microfluidic system (Fluxion Biosciences, South San Francisco, CA). Channels of a 48-well BioFlux plate were biofunctionalized for 3 hours at 37°C with either 2 % BSA serving as negative control or plasmatic VWF (100 µg/ml) and rinsed with 0.5% BSA. Channels were perfused for 10 min with different erythrocyte or artificial vesicle preparations at 2 dyne/cm² and 10 dyne/cm², respectively. Reflection interference contrast microscopy (RICM) was used to visualize the interactions of the erythrocytes with the coated channel surfaces.

Reflection Interference Contrast Microscopy (RICM). For RICM visualization, an inverted Microscope Observer Z1 (Zeiss, Jena, Germany) with a Colibri LED illumination system was used at two excitation wavelengths, 470 nm and 555 nm. Images were captured with a monochromatic camera (Axiocam MRm, Zeiss, Jena, Germany). The acquisition time was 200 ms at 350 ms intervals.

Endothelial cell preparation. Human Umbilical Vein Endothelial Cells (HUVEC) were isolated and cultivated as described previously [4, 5]. HUVEC were maintained at 37°C and 5% CO_2 and seeded into gelatine-coated microchannels after the second passage in accordance with the protocols recommended by Fluxion Biosciences.
HUVEC were cultured inside the flow channels using Endothelial Cell Growth Medium 2 (EGM-2, Lonza, Cologne, Germany) until confluence was achieved. Secretion of VWF was stimulated using 100 nM phorbol myristate acetate (PMA; Sigma-Aldrich, Taufkirchen, Germany) in EGM-2 for 10 min before starting the flow experiments.
Human Dermal Microvascular Endothelial Cells (HDMEC) were obtained from PromoCell (Heidelberg, Germany) and cultivated in IBIDI flow channels (IBIDI, Martinsried, Germany) using EGM-2 as described previously[5]. After reaching confluency 50 nM histamine was used to stimulate WPB exocytosis. Perfusion time was 10 min at 2 dyne/cm² before fixation with 4% PFA at 4°C.

Immunostaining flow experiments. To quantify the adherent erythrocytes in the VWF-coated channels, FITC-labeled Annexin-V-Fluos (Roche Diagnostics, Mannheim, Germany) was used during sample preparation. Immunostaining of the adherent erythrocytes on HUVECs and stimulated HDMECs was carried out as follows: fixation was performed with 4% paraformaldehyde (PFA) for 30 min at 4°C, followed by washing with 0.5% BSA and blocking with 2% BSA. The primary and secondary antibodies (1:200 in 0.5% BSA) were incubated for 45 min at room temperature. All steps were carried out at 2 dyne/cm² for 5 min in the flow direction used in the initial experiment. Images were captured immediately after a final rinsing step.
The following antibodies were used:
FITC-polyclonal sheep anti human VWF (GeneTex Inc., Irvine, CA, USA),
Monoclonal mouse anti human Band 3 (Sigma-Aldrich, Taufkirchen, Germany),
Texas red goat anti mouse (Invitrogen, Eugene, Oregon, USA).

Lipid vesicles. Giant unilamellar vesicles were prepared by electroformation as previously described [6]. The Texas Red DHPE (T-Red) lipids (Invitrogen; Carlsbad, CA, USA), 1,2-dimyristoyl-sn-glycero-3-phosphocholine (DMPC), 1,2-dimyristoyl-*sn*-glycero-3-phospho-L-serine (DMPS) and 1-palmitoyl-2-oleoyl-*sn*-glycero-3-phosphoethanolamine (POPE) (Avanti Polar Lipids, Alabaster, Alabama, USA) were mixed to the desired composition in chloroform/methanol (9:1) and deposited on indium tin oxide-coated glass slides. The remaining solvent was removed in a vacuum desiccator for 3 h. The slides were assembled in parallel in a chamber containing 290 mOsm sucrose solution. Vesicle formation was enhanced by an electrical AC field with a frequency of 15 Hz and an amplitude of 1.5 V mm^{-1} applied overnight.
Samples were prepared with the following relative compositions: 98:2 DMPC:T-Red, 88:10:2 DMPC:DMPS:T-Red and 88:10:2 DMPC:POPE:T-Red. The phase transition temperature of all lipid mixtures was well below the temperature of 37°C used during the experiment.

ELISA. To quantify VWF-AV binding, a modified sandwich ELISA technique using a polyclonal rabbit anti-VWF antibody (Dako, Copenhagen, Denmark) and a polyclonal rabbit peroxidase-labeled anti-human VWF antibody (Dako, Copenhagen, Denmark) was applied. Transparent 96-well plates were coated with either 20 µg/ml purified AV from human placenta (Sigma-Aldrich, Taufkirchen, Germany), 20 µg/ml PS or 2 µg/ml anti-VWF antibody to generate an immobilized substrate. Various concentrations (0.15, 0.5, 1.0 and 1.5 µg/ml) of human plasmatic VWF (Calbiochem, Bad Soden, Germany) were added, and adhesion was quantified colorimetrically. All washing steps were performed with calcium-free buffer (for AV) or detergent-free buffer (for PS).

CRF Patients. Five patients with CRF, mild anemia and erythropoietin substitution in a saturated state were included in the study. Informed consent was obtained from all subjects before inclusion. The study was conducted according to the ethical guidelines of our institution and the Helsinki Declaration. A 7.5 ml sample of heparin blood was taken from the CRF patients (n=5) and healthy donors (n=5) and prepared as described above.

Mouse experiments. All experimental protocols were conducted in accordance with the German law on animal protection and were approved by the Regierungspräsidium in Tübingen. Homozygous $VWF^{-/-}$ mice [7] (B6.129S2-Vwf^{tm1Wgr}/J) (Jackson Laboratory/Charles River, Sulzfeld, Germany) were compared with C57Bl6 control mice. Renal ischemia/reperfusion procedures were performed using 4- to 5-month old male $VWF^{-/-}$ and control mice as described [8]. Briefly, animals were anesthetized using a mixture of midazolam (5 mg/kg b.w.), medetomidine (0.5 mg/kg b.w.) and fentanyl (0.05 mg/kg b.w.) and placed on a controlled temperature heating pad (37°C). The right kidney was excised through an incision in the right flank. The left kidney was dissected from the surrounding tissue and placed in a lucite cup before being covered with a moist swab. The left renal artery was occluded by an 8-0 (Ethicon) suture in a hanging weight system. Following 45 minutes of ischemia, the kidney was reperfused by removal of the hanging weight and the filament. Reperfusion was confirmed visually. The incision was closed, and the animals were treated with buprenorphine for analgesia. After 24 h, the animals were sacrificed by being re-anaesthetized and perfused at a constant flow rate of 5 ml/min of PBS, followed by fixation in 4% PFA.

Morphological evaluation of renal histology. For histological analyses, the kidneys were perfused and fixed with 4 % PFA and then dehydrated in ethanol and xylol before being embedded in paraffin. Paraffin embedded tissues were cut into sections of 2 µm thickness and stained with periodic acid Schiff (PAS) and Sirius red stains. Renal morphology was investigated by light microscopy as described below, with the investigator blinded to the genotype of the mice. Tubulointerstitial damage, i.e., tubular necrosis, tubular atrophy and tubular dilation, was assessed in PAS-stained paraffin sections at a magnification of 200x using a semiquantitative scoring system (acute tubular necrosis [ATN] score, displayed in Supplementary Table 2) [9]. To determine the ATN score, 15 fields were randomly sampled per kidney and graded as follows: grade 0, no change; grade 1, necrosis involving less than 25% of the area; grade 2, necrosis affecting 25–50% of the area; grade 3, necrosis involving more than 50% of the area, and grade 4 necrosis involving almost the entire area. Erythrocyte attachment was monitored in the inner stripe (IS) of diseased kidneys in 12 fields at 200x magnification and graded using a 0-4 grading system (grade 0, no erythrocytes attached; grade 1, sporadic erythrocytes attached; grade 2, attached erythrocytes in more than 25% of the capillaries; grade 3, attached erythrocytes in more than 50% of the capillaries; grade 4, attached erythrocytes in more than 75% of the capillaries, displayed in Supplementary Table 1).

Localization of VWF after I/R. Localization of VWF within the injured kidney was investigated by immunofluorescence double staining using confocal laser scanning microscopy (Zeiss LSM 710 scanning unit equipped with an Argon, a HeNe 633 laser and a DPSS 561-10 laser on an Axio Observer Z1 inverted microscope Zeiss Plan-Acromat 63x/1,4 Oil DIC M27) at room temperature. Formalin fixed paraffin sections of 2 µm were boiled for antigen retrieval in a pressure cooker for 2,5 min using target retrieval

solution (DAKO, Glostrup, Denmark). After blocking with 1% BSA sections were incubated with polyclonal rabbit anti-human VWF (Dako, Copenhagen, Denmark) and monoclonal rat anti-mouse CD42b (GPIb, emfret Analytics, Eibelstadt, Germany) for 1 hour at 37°C, followed by washing steps and incubation with secondary donkey anti-rabbit IgG Alexa Fluor488 and donkey anti-rat Alexa Fluor594 (Life Technologies GmbH, Darmstadt, Germany). For signal enhancement, both primary as well as secondary antibodies were incubated in signal-enhancing solution (Pierce Immunostain Enhancer, Thermo Scientific Pierce Protein Research, Rockford, IL, USA). Before embedding the slides in moviol (Merck, Darmstadt, Germany), nuclei were stained with DAPI (Merck). Negative controls for immunostaining included either deletion or substitution of the primary antibody with equivalent concentrations of an irrelevant preimmune rabbit IgG. We used the acquisition software Zen 2009 (Carl Zeiss Microscopy GmbH) for image acquisition. No additional software was used to process the image data.

***In vivo* imaging of erythrocyte accumulation.** Mice were anesthetized by an intraperitoneal injection of ketamine (80 mg/kg; Ceva) and xylazine (14 mg/kg; Ceva), and surgical preparation of dorsal skinfold chambers (DSCs) was performed as described previously[10]. Mice were allowed to recover from surgery for 24 h.

For *in vivo* microscopic observations, the DSC was attached to the microscope stage, and intravital microscopy was performed using an epifluorescence microscope (AxioImager.Z2; Zeiss) with a 10x (numerical aperture 0.3) Plan-Neofluar magnification objective. Before IVM was performed in the DSC, 10^9 erythrocytes, extracted from wild-type donor mice, were treated with either normal or hyperosmolar Ringer solution, labeled with CFSE (20 μM) and intravenously transferred into wild-type recipient mice. Erythrocyte localization was recorded by attached cameras (AxioCam MRm/MRc; Zeiss), and data acquisition was performed with AxioVision software (version 4.8; Zeiss).

Fields of equally exposed microscopic images were analyzed for erythrocyte accumulation.

Statistical analyses. STATA12 for Windows was used for statistical analysis. Data are presented as the mean ± s.e.m. Two-sided tests were used throughout, and the differences were considered statistically significant at $P < 0.05$. Pairwise (univariate) comparisons were performed using Student's *t* test or the Mann-Whitney *U* test as appropriate.

Supplementary references

1. Nicolay JP, Schneider J, Niemoeller OM, Artunc F, Portero-Otin M, Haik G, Jr., Thornalley PJ, Schleicher E, Wieder T and Lang F. Stimulation of suicidal erythrocyte death by methylglyoxal. *Cell Physiol Biochem*. 2006;18:223-32.
2. Lang PA, Schenck M, Nicolay JP, Becker JU, Kempe DS, Lupescu A, Koka S, Eisele K, Klarl BA, Rubben H, Schmid KW, Mann K, Hildenbrand S, Hefter H, Huber SM, Wieder T, Erhardt A, Haussinger D, Gulbins E and Lang F. Liver cell death and anemia in Wilson disease involve acid sphingomyelinase and ceramide. *Nat Med*. 2007;13:164-70.
3. Schneppenheim R, Michiels JJ, Obser T, Oyen F, Pieconka A, Schneppenheim S, Will K, Zieger B and Budde U. A cluster of mutations in the D3 domain of von Willebrand factor correlates with a distinct subgroup of von Willebrand disease: type 2A/IIE. *Blood*. 2010;115:4894-901.
4. Schiviz A, Wuersch K, Piskernik C, Dietrich B, Hoellriegl W, Rottensteiner H, Scheiflinger F, Schwarz HP and Muchitsch EM. A new mouse model mimicking thrombotic thrombocytopenic purpura: correction of symptoms by recombinant human ADAMTS13. *Blood*. 2012;119:6128-35.
5. Pappelbaum KI, Gorzelanny C, Grassle S, Suckau J, Laschke MW, Bischoff M, Bauer C, Schorpp-Kistner M, Weidenmaier C, Schneppenheim R, Obser T, Sinha B and Schneider SW. Ultralarge von Willebrand factor fibers mediate luminal Staphylococcus aureus adhesion to an intact endothelial cell layer under shear stress. *Circulation*. 2013;128:50-9.
6. Angelova MI and Tsoneva I. Interactions of DNA with giant liposomes. *Chem Phys Lipids*. 1999;101:123-37.
7. Ni H, Denis CV, Subbarao S, Degen JL, Sato TN, Hynes RO and Wagner DD. Persistence of platelet thrombus formation in arterioles of mice lacking both von Willebrand factor and fibrinogen. *J Clin Invest*. 2000;106:385-92.
8. Grenz A, Eckle T, Zhang H, Huang DY, Wehrmann M, Kohle C, Unertl K, Osswald H and Eltzschig HK. Use of a hanging-weight system for isolated renal artery occlusion during ischemic preconditioning in mice. *Am J Physiol Renal Physiol*. 2007;292:F475-85.
9. Bernhardt WM, Campean V, Kany S, Jurgensen JS, Weidemann A, Warnecke C, Arend M, Klaus S, Gunzler V, Amann K, Willam C, Wiesener MS and Eckardt KU. Preconditional activation of hypoxia-inducible factors ameliorates ischemic acute renal failure. *J Am Soc Nephrol*. 2006;17:1970-8.
10. Hillgruber C, Poppelmann B, Weishaupt C, Steingraber AK, Wessel F, Berdel WE, Gessner JE, Ho-Tin-Noe B, Vestweber D and Goerge T. Blocking neutrophil diapedesis prevents hemorrhage during thrombocytopenia. *J Exp Med*. 2015;212:1255-66.

Supplementary Figures:

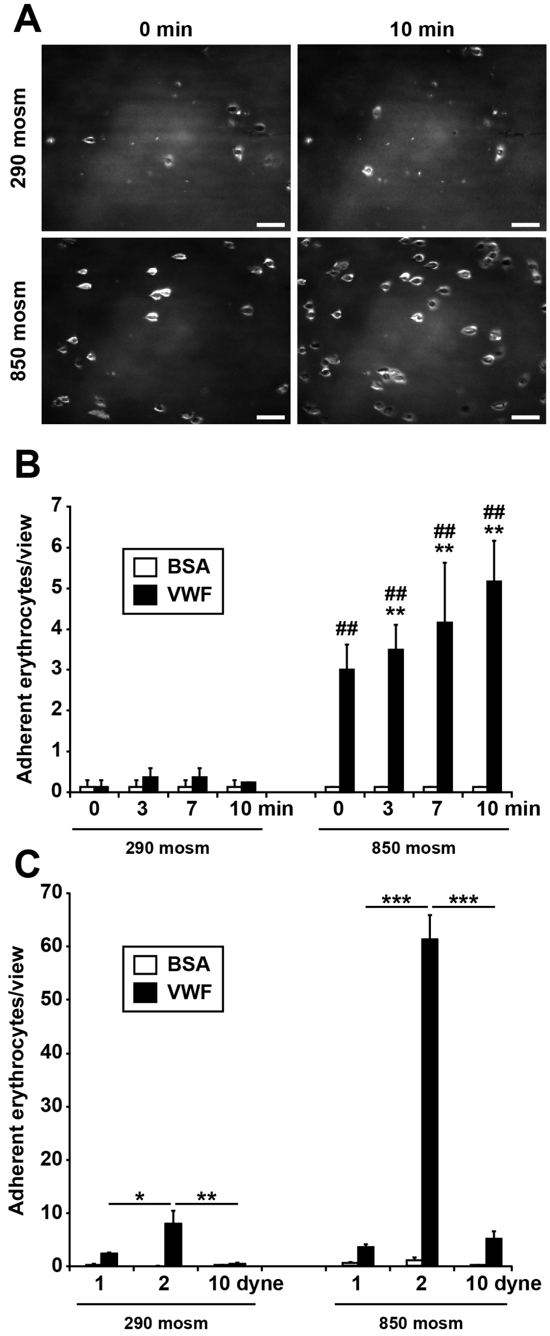

Supplementary Figure 1: Eryptotic erythrocytes bind to VWF at 10 dyne/cm^2. (A) original RICM pictures on VWF surface and **(B)** quantification of erythrocyte adherence in VWF- or BSA-coated flow channels visualized by RICM incubated for 6 hours in the indicated osmolarity after the indicated time at a flow rate of 10 dyne/cm^2 (n=4). **(C)** Comparison of erythrocyte adherence quantification in VWF- or BSA-coated flow channels at distinct shear rates of 1, 2 and 10 dyne/cm^2. ** means significance with $p<0.01$ to baseline control of the respective group at 0 min, as determined by Student's t test. ## means significance with $p<0.01$ of 850 mosm values compared to corresponding 290 mosm values (Student's t test). Scale bars correspond to 20 μm.

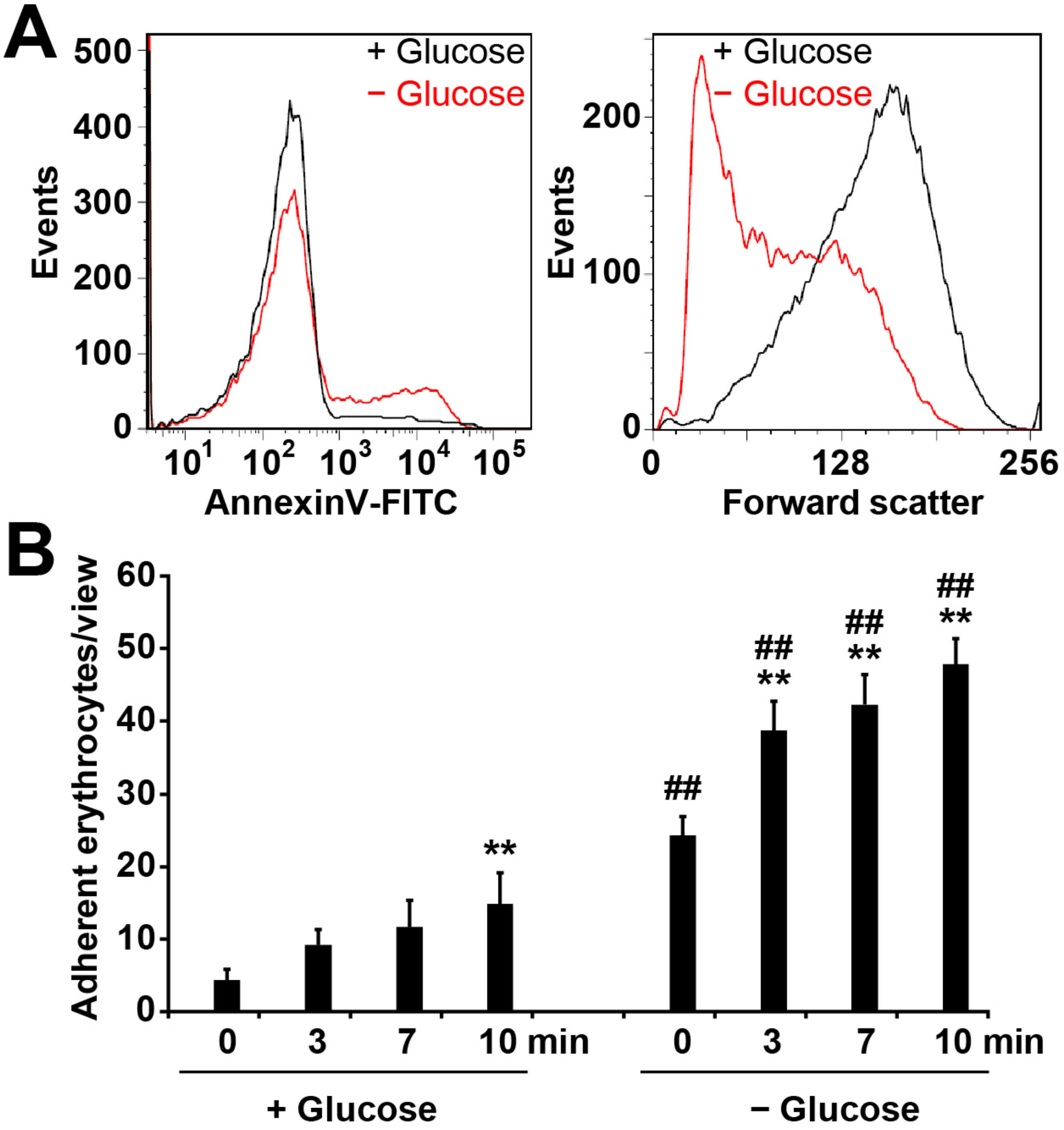

Supplementary Figure 2: Glucose depleted eryptotic erythrocytes bind to VWF. (A) FACS Annexin-V staining (left panel) and forward scatter (right panel) of unstressed (black curve) and eryptotic (red curve) erythrocytes after 48 hours of incubation in presence or absence of glucose. **(B)** Quantification of erythrocyte adherence in VWF-coated flow channels visualized by RICM incubated for 48 hours with and without glucose after the indicated time at a flow rate of 2 dyne/cm^2 (n=4). ** means significance with p<0.01 to baseline control of the respective group at 0 min, as determined by Student's t test. ## means significance with p<0.01 of glucose-free values compared to corresponding control values with glucose (Student's t test).

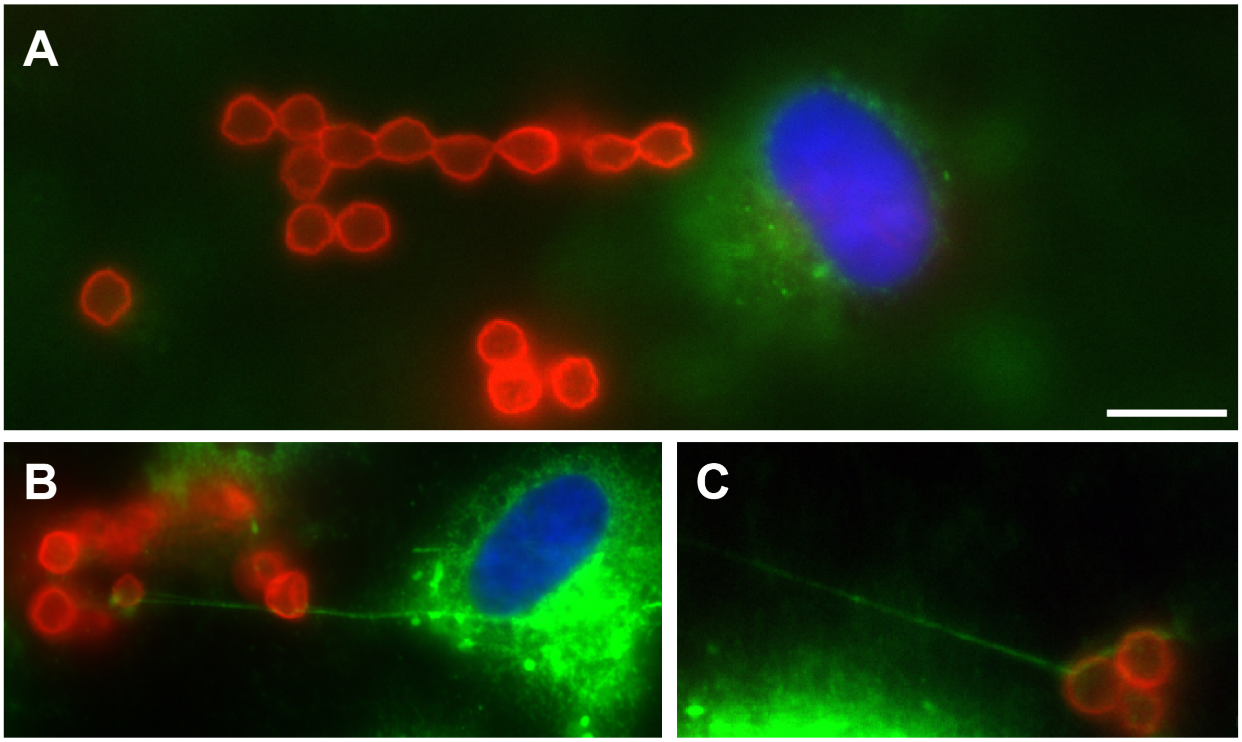

Supplementary Figure 3: ULVWF fibers from HUVEC bind eryptotic erythrocytes in a linear manner and in clusters. Eryptotic erythrocyte adherence and clustering to ULVWF fibers released by endothelial cells upon stimulation under distinct flow conditions (red: AE-1; green: VWF; blue: DAPI). Scale bar corresponds to 20 µm.

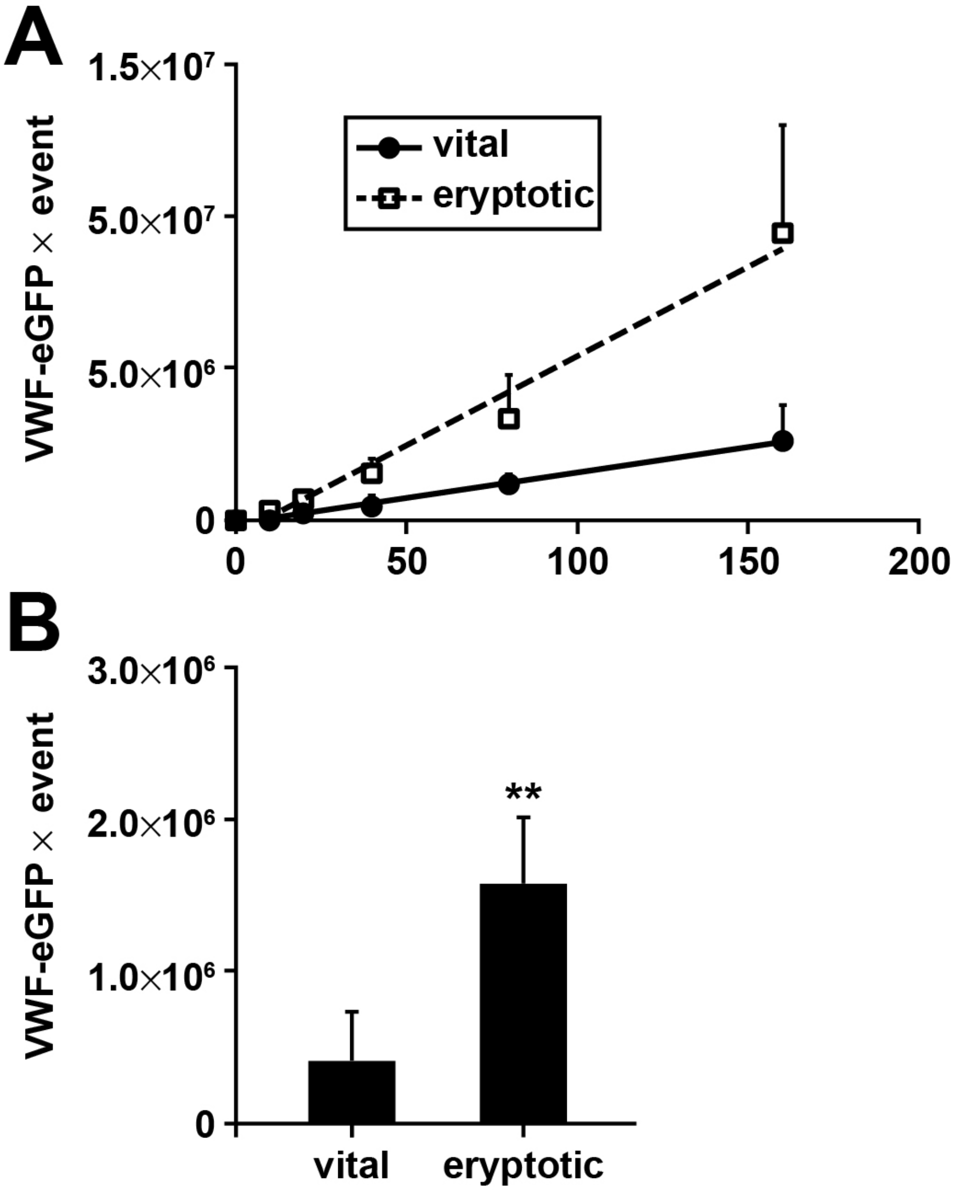

Supplementary Figure 4: Flow cytometric analysis of VWF-erythrocyte interaction. (A) Dose-dependent binding of eGFP tagged VWF to vital or eryptotic erythrocytes. Amount of erythrocyte-bound VWF as measured by the eGFP fluorescence signal was normalized to the number of measured erythrocytes (VWF-eGFP x events). Spearman rank correlation indicate a significant linear regression between the applied VWF concentration and the amount of bound VWF (n=3; P<0.01). (**B)** Association rate of VWF and Erythrocyte at a VWF concentration of 40µg/ml indicate a significantly increased VWF binding to eryptotic erythrocytes in comparison to vital erythrocytes (n=3). ** p<0.01 (Student's t test).

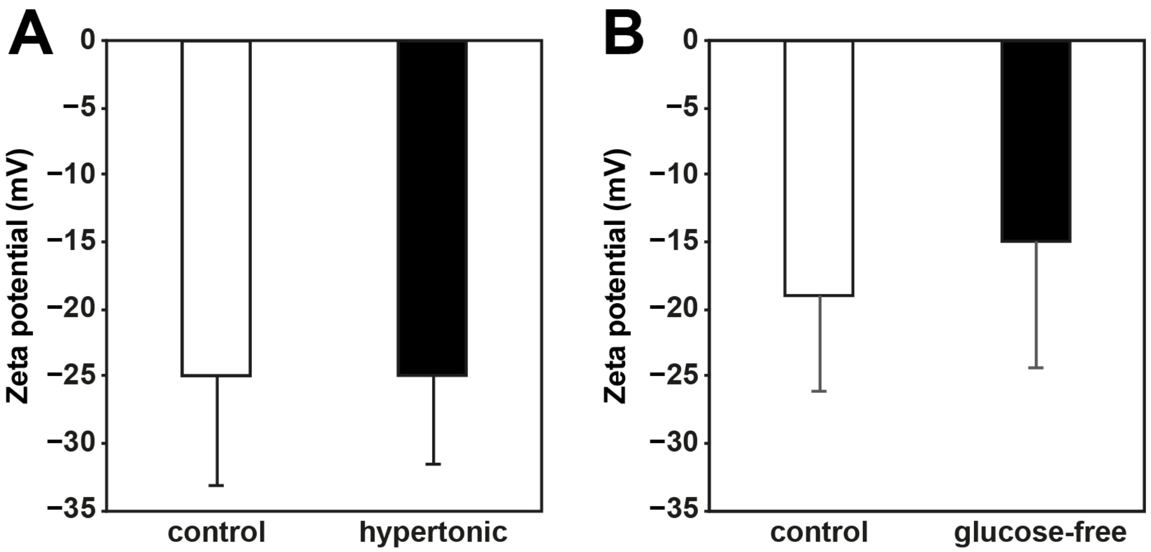

Supplementary Figure 5 Zeta potential of vital and stressed erytrocytes. Eryptosis was induced by incubation of erytrocytes in hypertonic solution for 6h **(A)** or through glucose depletion **(B)** for 24h. Corresponding control (control) erythrocytes were incubated in physiological Ringer solution (n=3).

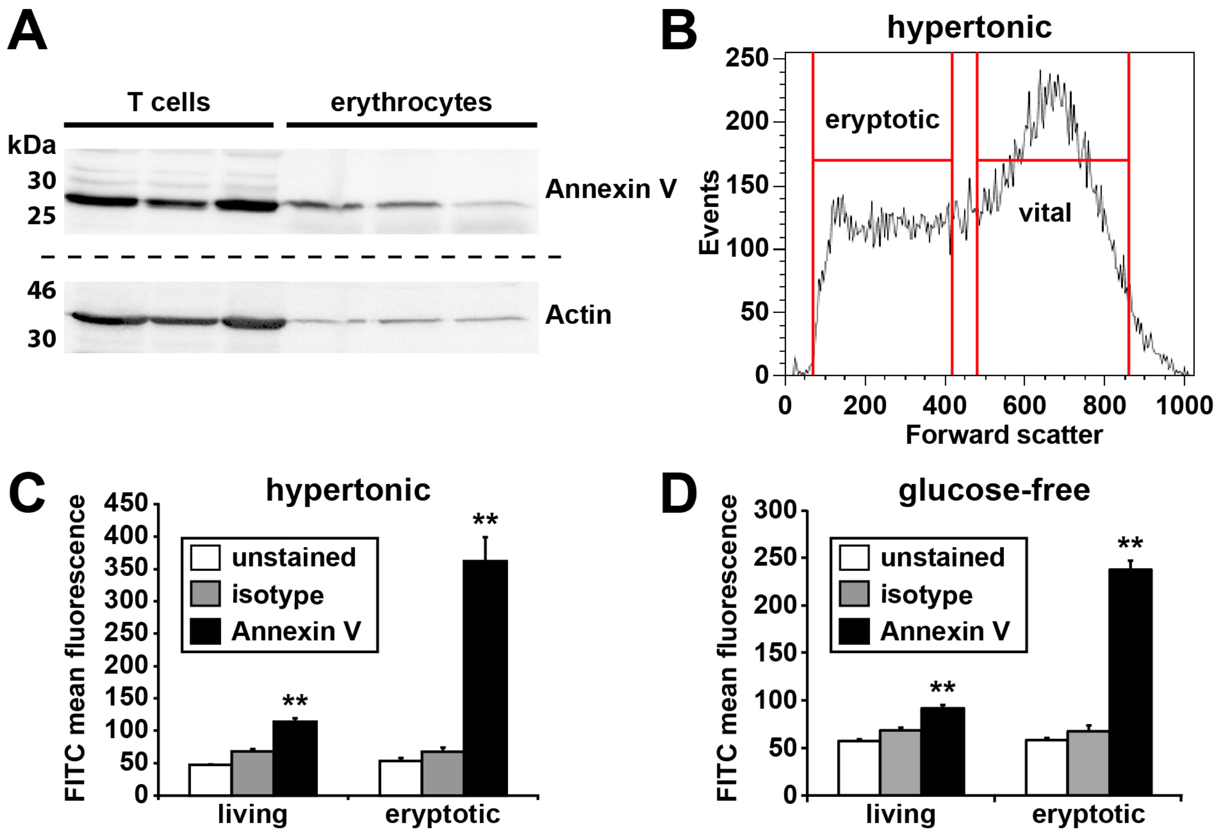

Supplementary Figure 6: Erythrocytic Annexin V increases VWF binding of eryptotic erythrocytes. **(A)** Western blot analysis of lysed primary human lymphocytes and erythrocytes from three healthy donors for the presence of Annexin V (see suppl. Fig. 8). **(B)**, **(C)**, FACS analysis of an erythrocyte suspension containing vital and eryptotic cells differentiated by cell volume via forward scatter analysis **(B)** stained against surface Annexin V (n=3) **(C)**. **(D)** FACS analysis of Annexin V on the surface of living and eryptotic erythrocytes in a glucose-depleted suspension. (n=3). **p<0.01, as determined by Student's t test.

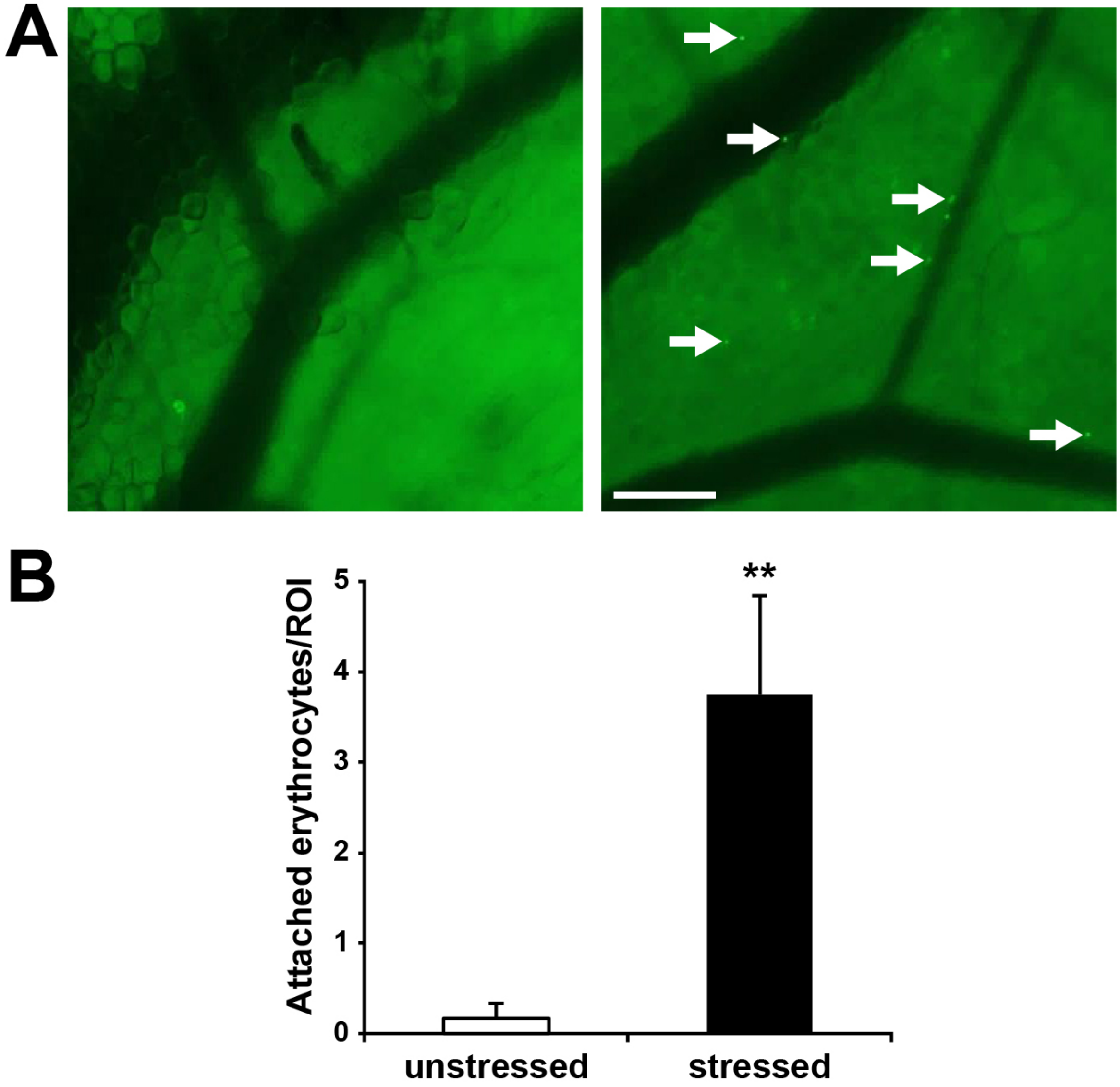

Supplementary Figure 7: *In vivo* imaging reveals localization of stressed erythrocytes in cutaneous blood vessels. (A) Representative microscopic images from the cutaneous microcirculation of wildtype mice equipped with a dorsal skinfold chamber (DSC) and transfused with CFSE-labeled normal (5-6 hours Ringer solution) or stressed (5-6 hours hypertonic Ringer solution) erythrocytes (white arrows). Scale bar = 100 µm. **(B)** Quantification of attached erythrocytes per view/region of interest (ROI) in the viewfield of the DSC. Data are presented as mean values ± SEM of n ≥ 3 mice per group of at least three individual experiments. ** means significance with $p<0.01$ of stressed erythrocyte adherence values compared to corresponding control values with unstressed erythrocytes (Student's t test).

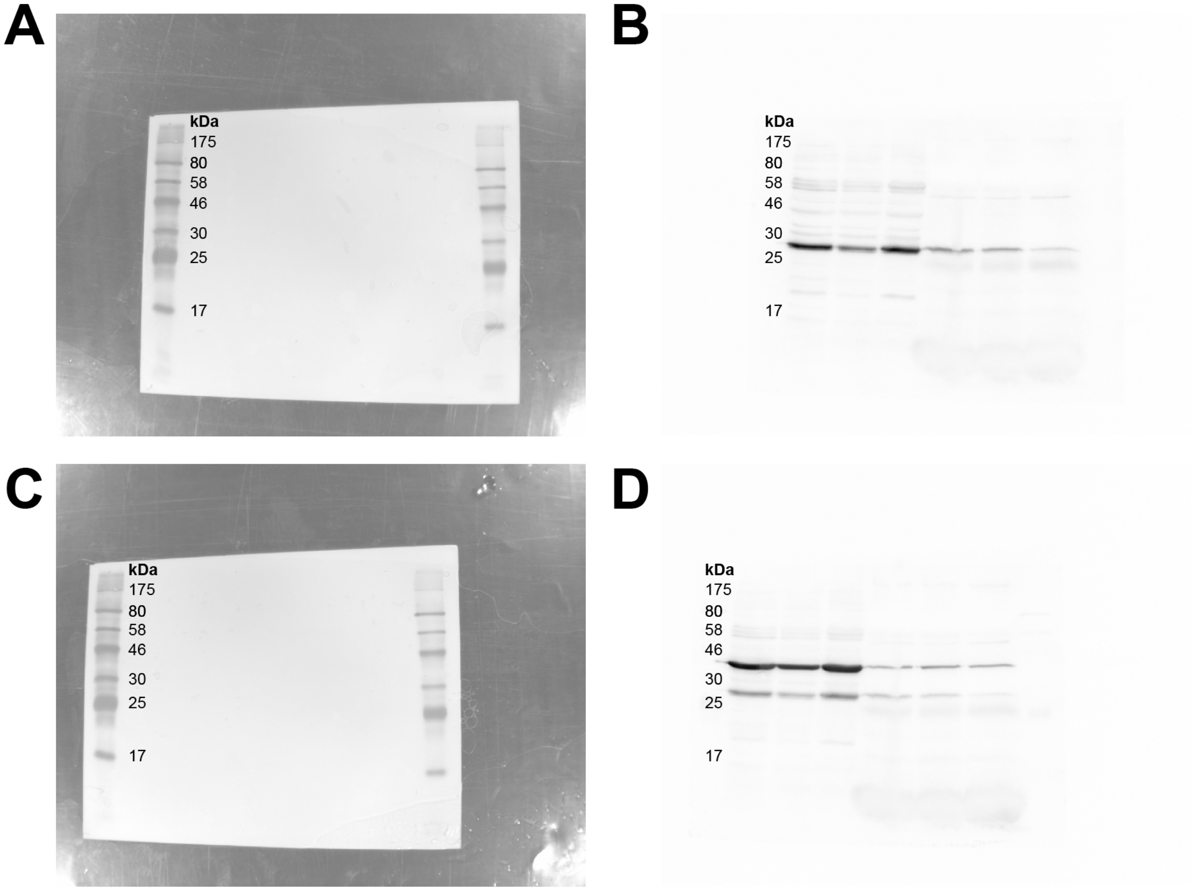

Supplementary Figure 8: Original data for Western blot analysis of Annexin V and Actin. Western blot analysis showing protein ladders **(A, C)** and protein bands for Annexin V **(B)** and Actin **(D)**.

Supplementary Tables:

Score	Feature
0	No visible erythrocytes
1	Single scattered erythrocytes
2	Erythrocytes in >25% of capillaries
3	Erythrocytes in >50% of capillaries
4	Erythrocytes in >75% of capillaries

Supplementary Table 1: Semiquantitative score for erythrocyte adhesion in renal capillaries

Score	Feature
0	Normal tubular morphology
1	Tubular dilatation in <25%
2	Tubular dilatation and atrophy in <50%
3	>50% dilated tubuli, atrophy, necrosis, protein casts
4	>75% dilated tubuli, atrophy, necrosis, protein casts

Supplementary Table 2: Semiquantitative score for acute necrosis of renal tubuli as a histological readout for renal damage

Supplementary Videos:

Supplementary videos 1-4: Characterization of dynamic interactions of stressed erythrocytes with activated endothelium.
Four representative video sequences of either unstressed (videos 1 and 2) or stressed (videos 3 and 4; pre-incubation 6 hours in hypertonic solution) erythrocytes in either unstimulated (videos 1 and 3) or histamine-stimulated (videos 2 and 4) HUVEC-coated flow channels (10 minutes; flow rate of 1.5 dyne/cm^2) visualized by RICM. Scale bars correspond to 20 µm. (n=4 each).

Supplementary video 5: Distinct characteristics of transient interactions and adhesion events of stressed erythrocytes on activated endothelial cells. Z-axis-oversubscribed three-dimensional RICM reconstruction video sequence of hypertonic stressed erythrocytes superfusing activated endothelial cells at 1.5 dyne/cm^2 with the focal plane at the microfluidic luminal endothelial cell surface. The video was taken with a framerate of 5 fps. Erythrocytes are visualized as white (flat) or red (elevated) annuli. Transient interactions and prolonged adhesion events are observable.

Supplementary video 6: Eryptotic erythrocyte binding to endothelial derived ULVWF controlled by Annexin V. RICM movies of hypertonic stimulated (850 mosm) dynamic erythrocyte-ULVWF interactions (co) supplemented with Annexin V, an anti-Annexin V antibody or heparin as indicated for 60 real-time seconds after 10 minutes of flow at 1.5 dyne/cm^2 (n=4 each). The videos were taken with a framerate of 5 fps and played with 15 fps. Scale bars correspond to 20 µm.
